# Alleviation of Nitrogen and Sulfur Deficiency and Enhancement of Photosynthesis in *Arabidopsis thaliana* by Overexpression of Uroporphyrinogen III Methyltransferase (*UPM1*)

**DOI:** 10.3389/fpls.2017.02265

**Published:** 2018-01-23

**Authors:** Sampurna Garai, Baishnab C. Tripathy

**Affiliations:** School of Life Sciences, Jawaharlal Nehru University, New Delhi, India

**Keywords:** carbon assimilation, electron transport, nitrogen utilization efficiency, nitrogen deficiency photosynthesis, siroheme, sulfur deficiency, uroporphyrinogen III methyltransferase1 (UPM1)

## Abstract

Siroheme, an iron-containing tetrapyrrole, is the prosthetic group of nitrite reductase (NiR) and sulfite reductase (SiR); it is synthesized from uroporphyrinogen III, an intermediate of chlorophyll biosynthesis, and is required for nitrogen (N) and sulfur (S) assimilation. Further, uroporphyrinogen III methyltransferase (UPM1), responsible for two methylation reactions to form dihydrosirohydrochlorin, diverts uroporphyrinogen III from the chlorophyll biosynthesis pathway toward siroheme synthesis. *AtUPM1* [At5g40850] was used to produce both sense and antisense plants of *Arabidopsis thaliana* in order to modulate siroheme biosynthesis. In our experiments, overexpression of *AtUPM1* signaled higher NiR (*NII*) and *SiR* gene and gene product expression. Increased *NII* expression was found to regulate and enhance the transcript and protein abundance of nitrate reductase (NR). We suggest that elevated NiR, NR, and SiR expression must have contributed to the increased synthesis of S containing amino acids in *AtUPM1*overexpressors, observed in our studies. We note that due to higher N and S assimilation in these plants, total protein content had increased in these plants. Consequently, chlorophyll biosynthesis increased in these sense plants. Higher chlorophyll and protein content of plants upregulated photosynthetic electron transport and carbon assimilation in the sense plants. Further, we have observed increased plant biomass in these plants, and this must have been due to increased N, S, and C assimilation. On the other hand, in the antisense plants, the transcript abundance, and protein content of NiR, and SiR was shown to decrease, resulting in reduced total protein and chlorophyll content. This led to a decrease in photosynthetic electron transport rate, carbon assimilation and plant biomass in these antisense plants. Under nitrogen or sulfur starvation conditions, the overexpressors had higher protein content and photosynthetic electron transport rate than the wild type (WT). Conversely, the antisense plants had lower protein content and photosynthetic efficiency in N-deficient environment. Our results clearly demonstrate that upregulation of siroheme biosynthesis leads to increased nitrogen and sulfur assimilation, and this imparts tolerance to nitrogen and sulfur deficiency in *Arabidopsis thaliana* plants.

## Introduction

The metabolic pathways of carbon, nitrogen and sulfur are intertwined with each other, leading to interdependence. Carbon metabolism includes reactions in both photosynthesis and respiration, whereas nitrogen metabolism, particularly in non-leguminous plants, involves assimilation of nitrate (${\text{NO}}_{3}^{-}$) from the soil. The supply of soil nitrogen is usually the limiting factor in the yield of plants in most agricultural systems (Robertson and Vitousek, 2009). Development of crops with low N requirement is expected to be extremely beneficial in addressing the environmental issues and commercial concerns related to the use of chemical fertilizers (Foyer and Ferrario, 1994; Robertson and Vitousek, 2009; Sutton et al., 2011).

After its uptake from the soil, ${\text{NO}}_{3}^{-}$ is reduced to ${\text{NH}}_{4}^{+}$, using nitrate reductase (NR) and nitrite reductase (NiR). NR is a multi-domain enzyme, which has, as prosthetic groups, molybdenum, Fe-heme and FAD in a 1:1:1 stoichiometry. The NR mediates electron transfer from NAD(P)H to nitrate, reducing it to ${\text{NO}}_{2}^{-}$ (Campbell, 1999). Further, the assimilation of ${\text{NO}}_{2}^{-}$, thus formed, by NiR is essentially performed by its prosthetic group siroheme (Murphy et al., 1974) that catalyzes 6 electron reduction of ${\text{NO}}_{2}^{-}$ to ${\text{NH}}_{4}^{+}$. Siroheme, in turn, is synthesized from uroporphyrinogen III, an intermediate of the biosynthetic pathway of chlorophyll (Chl) (Tanaka and Tanaka, 2007).

Animals are incapable of reducing sulfate and, thus, they require S-containing amino acids or proteins from plant sources. Therefore, sulfate assimilation by plants plays a pivotal role in the sustenance of human life (Tripathy et al., 2010). Plants take up sulfur from the soil, primarily as sulfate. In a plant cell, sulfate is reduced to sulfite by a series of reactions. Sulfite reductase (SiR), located in the plastids, is responsible for six electron reduction of ${\text{SO}}_{3}^{2-}$ to S^2−^ (Murphy et al., 1974) that requires siroheme as the prosthetic group.

In addition to siroheme, both the nitrite reductase and the sulfite reductase have a prosthetic group, which is [4Fe-4S] (Crane and Getzoff, 1996). Siroheme is an iron-containing tetrapyrrole, synthesized in the chloroplast (Murphy and Siegel, 1973); it bridges the iron sulfur cluster and the cysteine residue of the protein, and is involved in the reduction of nitrite and sulfite to ammonia and sulfide (Balk and Schaedler, 2014). Thus, we expect that endogenous levels of the available siroheme and the iron sulfur cluster would play a regulatory role in the metabolic reprogramming of N and S assimilation pathways.

**Figure 1:**
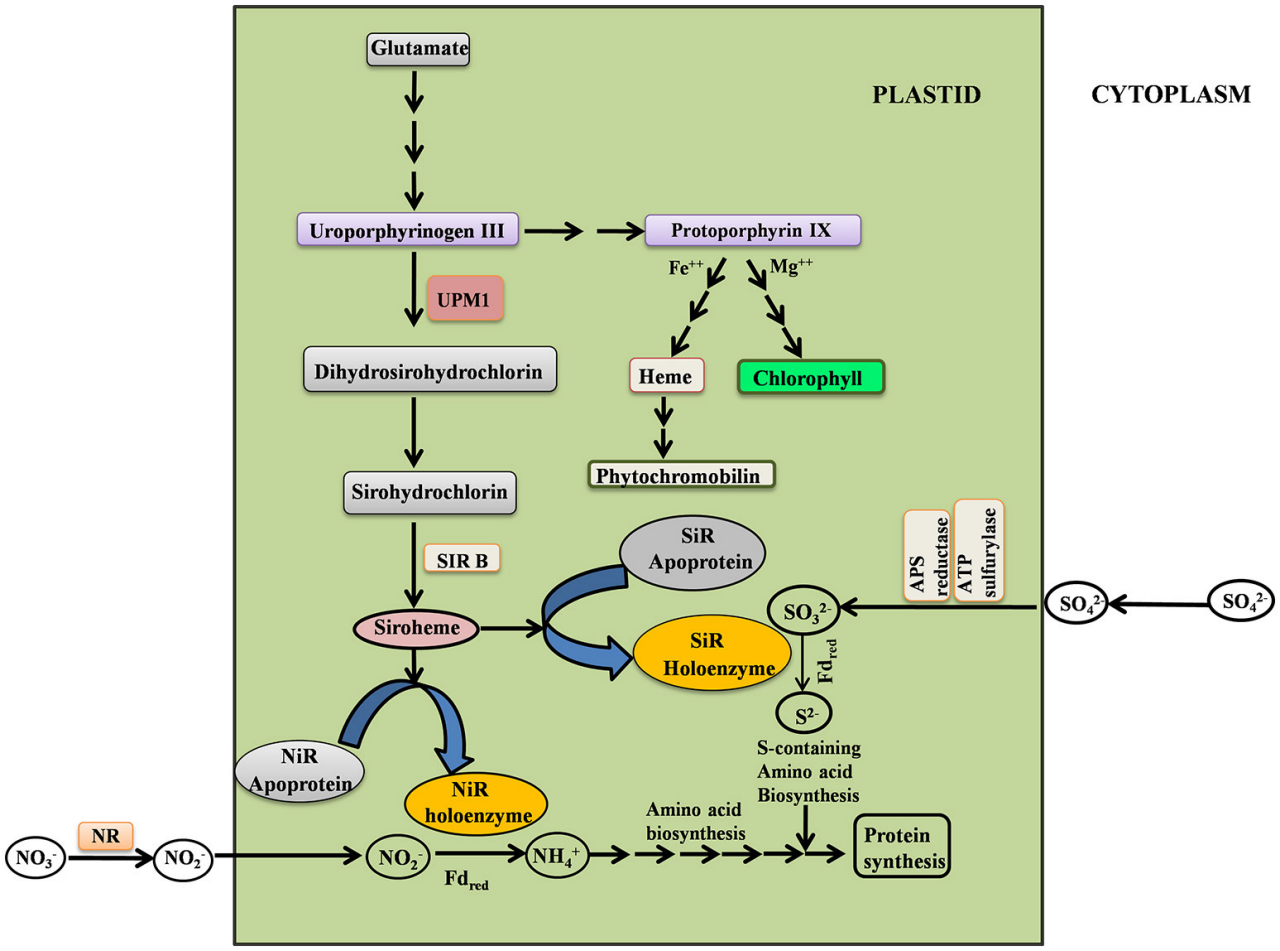
Simplified scheme depicting the interrelationship between siroheme, chlorophyll, heme biosynthesis and N & S assimilation. UPM1, Uroporphyrinogen III methyl transferase; SIRB, Sirohydrochlorin Ferrochelatase; NR, Nitrate reductase; NiR, Nitrite reductase; SiR, Sulfite reductase.

Siroheme biosynthesis is dependent on the synthesis of uroporphyrinogen III (Urogen III), a light sensitive compound; it is the first closed tetrapyrrole intermediate of the Chl biosynthesis pathway (Figure 1). Thus, from the Urogen III branch point of tetrapyrrole biosynthesis, siroheme is synthesized via two methylation steps involving oxidation and ferrochelation (Warren et al., 1990; Leustek et al., 1997; Tripathy et al., 2010). The enzyme uroporphyrinogen III methyltransferase (UPM1) catalyzes methylation at the C2 and C7 positions of Uroporphrinogen III to form dihydrosirohydrochlorin (Warren et al., 1990; Leustek et al., 1997): this reaction is a rate limiting step. The dihydrosirohydrochlorin undergoes oxidation to form sirohydrochlorin. The latter is converted to siroheme by sirohydrochlorin ferrochelatase (SIRB) that catalyzes the chelation of iron into the macrocycle (Raux-Deery et al., 2005). We note that the siroheme biosynthesis pathway is conserved in bacteria, yeast and higher plants. In bacteria, sirohydrochlorin cobalt chelatase inserts cobalt into sirohydrochlorin leading to vitamin B_12_ biosynthesis (Brindley et al., 2003). Heme is crucial for many biological processes and there are reports for the existence of pathways for the conversion of siroheme to heme in some denitrifying and sulfur reducing bacteria (Bali et al., 2011, 2014). Thus, the enzyme UPM1 is crucial for many essential biological processes.

All the three enzymes, NiR, NR, and SiR, are regulated by light via a phytochrome-mediated signaling cascade (Faure et al., 1991). The promoters of *SiR, NII*, and *NIA2* have light responsive elements (LRE). Interestingly, both siroheme biosynthetic genes i.e., *UPM1* and *SIRB* are also upregulated by light and have LRE in their promoters for interaction with the phytochromes (Garai et al., 2016). This demonstrates the pivotal role of light in the regulation of most of the N and S assimilation pathway.

In *Arabidopsis thaliana*, uroporphyrinogen III methyl transferase is coded by *UPM1* [At5g40850]. Further, AtUPM1 is a nuclear-encoded plastidic enzyme containing a transit peptide of 28 amino acids (Leustek et al., 1997). It is well known that the assimilation of N and S in plants is regulated by siroheme (Tripathy et al., 2010), and that the knockout lines for *AtUPM1* in *A. thaliana* are lethal (Tripathy et al., 2010; Saha et al., 2012). In the present study, we have analyzed the impact of over-expression or reduced expression of *AtUPM1* on N, S and C assimilation. We demonstrate here, *in A. thaliana*, that overexpression of *AtUPM1* enhances N and S assimilation, protein content and photosynthesis rates. Further, we demonstrate that overexpression of *AtUPM1* protects plants from nitrogen and sulfur deficiency.

## Materials and methods

### Plant material and growth conditions

*A. thaliana* seeds were soaked in water and kept for 48 h at 4°C (i.e., they were stratified). Seedlings were germinated and grown, at 21°C, in agropeat:vermiculite mixture (4:1) in pots (10-20 per pot under cool-white-fluorescent light (80 μmol photons m^−2^ s^−1^), and a 14 h light/10 h dark photoperiod.We also grew plants under sterile conditions for some experiments. For this, the seeds were sterilized and plated on half strength Murashige & Skoog (MS) media for 10 d (Murashige and Skoog, 1962). These seedlings were then transferred to half-strength MS plates, one plant per plate. In addition, we grew plants under nutrient deficient conditions. For this, *A. thaliana* seeds were grown in half-strength MS plates for 10 days and subsequently transferred to N-deficient or S-deficient growth media, and grown for additional 15 days. Here again, each plate contained one plant. We also grew wild type (WT) and transgenic plants directly on nutrient deficient media (see below for details) in order to have plants to induce severe N or S deficiency. For a proper comparison, both WT and transgenic plants were grown on a single square plate.

Growth media were prepared, as described below. We used Hoagland medium (Hoagland and Arnon, 1950), which had the following composition: (solution I) KH_2_PO_4_ (1 M); (solution II) MgSO_4_.7H_2_O (1 M); (solution III)*:* Ca(NO_3_)_2_.4H_2_O (1 M); (solution IV): Ferric citrate (0.5%); (solution V): H_3_BO_3_ (0.05%), MnSO_4_.7H_2_O (0.05%), ZnSO_4_.7H_2_O (0.005%), CuSO_4_.7H_2_O (0.002%), and Na_2_MoO_4_.2H_2_O (0.001%). One liter of the growth medium contained 1% sucrose and 1, 2, 5, 1, and 1 ml each of solutions I, II, III, IV, and V, respectively. The pH of the media was 5.7 with 0.6% agar as a solidifying agent. For the nitrogen-deficient media, we simply replaced Ca(NO_3_)_2_ with CaCl_2_, and for the sulfur deficient media, we replaced MgSO_4_ with MgCl_2_.

### qRT-PCR

The total RNA was isolated from the plant samples using Tri reagent (Sigma) from 3 different plants (i.e., three biological replicates). The first strand cDNA was synthesized using cDNA synthesis kit (Verso, USA). Relative expression of different genes was studied by performing qRT-PCR on an ABI Prism 7500 Sequence Detection System using the default program (Applied Biosystems, USA). Three technical replicates were taken per sample. We used 10 μl reaction mixtures, which contained 0.5 μl cDNA, 5 pmol primers (see Table 4) and 5 μl 2xSYBR Green PCR Master Mix (Applied Biosystems, USA). The reference gene used was *Actin 2*. The relative gene expression data were analyzed using 2^−ΔΔCt^ quantitation methods (Livak and Schmittgen, 2001).

### Cloning of *AtUPM1* cDNA in binary vector and transformation of *Arabidopsis thaliana*

The AtUPM1 coding DNA sequence (CDS) (accession # NM_123450) of the complete *AtUPM1* gene (At5g40850), consisting of 1,110 bp, was amplified from the cDNA library of WT *A. thaliana* (Col 0), using the gene specific primer pair: F-5′ATGGCTCTTGTTCAGCGGATTC3′ and R - 5′CTACCGGGTCTCTACAA GGCA 3′; it was then cloned into pGEMT-Easy vector (Promega corporation, USA). The pGEMT-Easy recombinant plasmid, containing the full length *At*UPM1 cDNA, was digested with EcoRI and then cloned into the modified pCAMBIA 1304 binary vector at the EcoRI site in both the sense and the antisense orientation, under the control of CaMV35S promoter (Supplementary Figure 1). The pCAMBIA 1304 binary vector was modified as described earlier (Pattanayak et al., 2005) and hygromycin-resistance (*hpt*) marker gene was replaced with kanamycin-resistance (*nptII*) gene. The pCAMBIA1304::*AtUPM1* construct was introduced into *Agrobacterium tumefaciens* strain GV3101 for the transformation of *A. thaliana* (Col-0) by the floral dip method and vacuum infiltration (Clough and Bent, 1998). The seeds of the transformed plants (the T1 generation) were screened on half-strength MS agar medium containing 50 mg l^−1^ kanamycin and were then grown up to the T4 generation. We used these T4 homozygous lines for all the studies reported in this paper.

### PCR

PCR was carried out with the genomic DNA of WT and transgenic plants serving as the template. The primers used were: 35S Int F: 5′ CCC ACT ATC CTT CGC AAG AC 3′ and *AtUPM1* R: 5′ CTACCGGGTCTCTACAAGGCA 3′ for the PCR confirmation of the sense plants; the positive plants yielded an amplicon of 1.3 kb (Supplementary Figure 2A). For the PCR confirmation of antisense plants, the primers used were: 35S Int F: 5′ CCC ACT ATC CTT CGC AAG AC and *AtUPM1* F: ATGGCTCTTGTTCAGCGGATTC; these yielded an amplicon of 1.3 kb for the positive antisense plants (Supplementary Figure 2B).

### Southern blot analysis of the transgenic plants

The presence of *AtUPM1* transgene was analyzed by Southern blot analysis. Thirty micrograms (μg) of genomic DNA from leaves of the T3 generation WT and transgenic plants (control and transgenic lines 4, 6, 7, and 9) were digested overnight, with XbaI enzyme. The digested DNA was resolved on 0.7% agarose gel and blotted onto Nylon 66 (MDI, USA) membrane. PCR was carried out using *AtUPM1* F for the preparation of the probe, and was labeled with [α^32^P] dCTP, using a radioactive random primer labeling kit (Amersham-GE, UK). The Southern blot was developed according to Sambrook and Russell (2001). All further analyses were carried out on homozygous lines of T4 through T7 generations.

### Protein and pigment estimation

The soluble protein, from the leaves, was estimated as described previously (Bradford, 1976). Ten biological replicates were taken for the analysis reported here. Total chlorophyll was extracted, from leaves, in 90% ammoniacal acetone, and estimated as described previously (Porra et al., 1989), whereas, the carotenoid content was also determined, as described previously (Wellburn and Lichtenthaler, 1984).

### Western blot analysis

Crude protein extracts were prepared from 3-week-old leaves for immunoblot analysis (Jilani et al., 1996). Total protein was resolved on a 12.5% SDS-PAGE (Laemmli, 1970), which was then transferred onto nitrocellulose membranes for western blot analysis. Polyclonal antibodies raised against *Arabidopsis thaliana* proteins were used for protein blot analyses of UPM1 (1:2,000), NiR (1:2,500), and NR (1:1,000). The bands in the immunoblots were evaluated using AlphaEase FC software.

### Nitrate reductase and nitrite reductase assays

Nitrate and nitrite reductase activities were measured, as described previously (Kaiser and Lewis, 1984; Takahashi et al., 2001).

### Pulse amplitude modulation (PAM) measurements of Chl a fluorescence

Chl *a* fluorescence measurement is used as a non-destructive and non-invasive signature of photosynthesis (Govindjee et al., 1986; Baker, 2008). The fluorescence from the ventral side of the leaf was measured with PAM-2100 chlorophyll fluorometer (Walz, Germany) at room temperature (Dutta et al., 2009). Leaves were dark-adapted for 20 min before fluorescence measurements (Demmig et al., 1987). The initial minimal fluorescence (Fo) was recorded by turning on a weak measuring beam set to a frequency of 0.6 kHz. The maximum fluorescence (Fm) was measured after a red (λ_650_ nm) saturation flash (3,000 μmol photons m^−2^ s^−1^) was given. The quantum efficiency of PSII in dark-adapted leaves was estimated as Fv/Fm (Fv = Fm-Fo) (Schreiber and Armond, 1978).

Electron transport rate (ETR) was estimated from the following relation: Yield × PAR × 0.5 × 0.84, where Yield is the overall photochemical quantum yield (inferred from (*F'*_*m*_ - *F*_*t*_)/*F'*_*m*_ = ΔF/*F'*_*m*_, with *F*_*t*_ being the measured fluorescence yield at any given time (t) and *F'*_*m*_ the maximal fluorescence yield in a pulse of saturating light when the sample is pre-illuminated); PAR is photon flux density of incident photochemically active radiation (μmol photons m^−2^ s^−1^); the 0.5 factor is used because the absorbed light energy is almost equally distributed between the two photosystems, whereas, 0.84 denotes that 84% of the incident quanta are absorbed by the leaf. The non-photochemical quenching (NPQ) of the excited state of Chl *a* was calculated as (F_m_ – F'_m_)/F'_m_ (Schreiber, 2004).

### Photosynthesis light-response curve

Light-response curve for photosynthesis of WT and *AtUPM1x* plants, grown for 5 weeks, in an agropeat:vermiculite (3:1) mixture, under 80 μmol photons m^−2^ s^−1^ light, was measured using an infrared gas analyzer GFS-3000 (Walz, Germany). Sample-chamber CO_2_ concentration was maintained at 400 μl L^−1^. The relative humidity was set at 70%. Air temperature, in the sample chamber, was maintained at 25°C. Leaves were pre-exposed for 20 min at 200 μmol photons m^−2^ s^−1^ before CO_2_ assimilation was monitored. Net CO_2_ assimilation was measured at several different light intensities.

### Porphobilinogen synthase (PBGS) assay

Leaves were collected from 25-day-old WT, AtUPM1x and antiUPM1 *A. thaliana* plants grown at 21°C, under cool-white-fluorescent light (80 μmol photons m^−2^ s^−1^) in a 14 h light/10 h dark photoperiod. The leaves (250 mg) were hand homogenized in 5 mL of 0.1 M Tris (pH 7.6) and 0.01 M β-mercaptoethanol solution at 4°C. The homogenate was centrifuged at 12,000 ^*^ g for 10 min at 4°C. The supernatant was used for enzymatic assays. The PBGS enzyme activity was determined by measuring the amount of PBG formed from ALA, as described earlier (Sood et al., 2005). PBG formed was calculated using the absorption coefficient of 6.2 ^*^ 10^4^ M^−1^ cm^−1^, at 555 nm (Hukmani and Tripathy, 1994).

### Porphobilinogen deaminase (PBGD) assay

Leaves (250 mg) collected from the WT, *AtUPM1x* and *antiUPM1 A. thaliana* plants, grown under cool-white-fluorescent light (80 μmol photons m^−2^ s^−1^) under a 14 h light/10 h dark photoperiod at 21°C, were hand homogenized in 5 ml of 0.1 M Tris (pH 7.6) and 0.01 M β-mercaptoethanol solution at 4°C. The homogenate was centrifuged at 10,000 rpm for 10 min at 4°C. The enzyme activity was assayed, using the supernatant; the amount of porphyrin synthesized in 1.0 ml of the reaction mixture was calculated, using the extinction coefficient of 5.48 × 10^5^ M^−1^ cm^−1^ at 405 nm (Bogorad, 1962).

### Amino acid estimation

Cysteine content was estimated as described earlier (Gaitonde, 1967). Fresh leaves were homogenized in 5% (w/v) ice-cold perchloric acid, and then centrifuged, for 1 h, at 3,000 g at 4°C. The supernatant was filtered through Whatman No. 1 filter paper. The filtrate was treated with acid ninhydrin reagent. The absorbance was read at 580 nm and the amount of cysteine was calculated with reference to the calibration curve obtained under similar conditions for standard cysteine.

The methionine content was estimated as described earlier (Horn et al., 1946). Fresh leaf samples (about 0.5 g) were refluxed with 6.0 mL 2.0 N HCl for 1 h, followed by evaporation on water bath with the addition of 1.0 g of activated charcoal. The filtrate was collected and the pH was adjusted to be 6.5 with the addition of 10 N NaOH. The volume was made upto 50 ml with the addition of distilled water. To 25 ml of this solution, 3 ml of 10% NaOH, 0.15 ml of 10% sodium nitroprusside, 1 ml of glycine solution (3%) and 2 ml of phosphoric acid were added in the order mentioned. This suspension was vigorously shaken and absorbance changes were measured at 520 nm to calculate the methionine concentration in the sample.

### Transmission electron microscopy

Leaves of *A. thaliana* transgenic plants were vacuum infiltrated with 2.5% glutaraldehyde solution for 30 min and kept overnight in the same solution (Karnovsky, 1965). The above solution was replaced by 0.1 M sodium-phosphate buffer (pH 7.0), and then we followed the method described by Jiang et al. (2011). Leaf sections were viewed under a Transmission Electron Microscope (JEOL 2100F).

### Statistical analyses

Microsoft Excel was used for the statistical analysis. The number of biological replicates taken for each experiment is noted in the figure legends. After the calculation of averages, standard deviations or standard errors for each of the parameters were determined and an unpaired Student's *t*-test was used to assess the difference between the WT and the transgenic plants, grown under different conditions.

## Results

### Transformation of *AtUPM1*cDNA in *Arabidopsis thaliana* and transgenic screening

The CDS region (accession no. NM_123450), consisting of 1110 base pair, was amplified from the cDNA library of WT *A. thaliana* (Col 0), as described in details under the Material and Methods. Confirmation of the transgenics, as well as of the copy number of the integrated gene, was checked by Southern blot analysis. Besides the endogenous gene of the WT, S4 and S6 transgenic lines had double insertion of the transgene, whereas, the S7 and S9 lines had single copy integration. The lines labeled as antisense “a” (ASa)and antisense “d” (Asd) had single copy integration, while that labeled as antisense “f” had a copy number of four (Supplementary Figure 2C).

The selected homozygous transgenic (T4) lines were analyzed in the leaves, by quantitative RT-PCR, for the abundance of *AtUPM1* message. The highest gene expression was found in the S7 line, which had a 3.5-fold higher transcript abundance than the WT. The other transgenic lines, i.e., S9, S6, and S4, respectively, had 2.4, 2.2, and 1.3 fold higher expression of *UPM1*. However, in the antisense lines, there was a decrease in the expression of *AtUPM1* message by 12–47% (Supplementary Figure 2D).

The overexpression and underexpression of the gene product in the transgenics was monitored by immunoblot analysis. Similar to transcript abundance, the AtUPM1 protein expression was higher (62–72%) in S7 and S9 lines than in the WT. On the contrary, antisense lines ASa, ASd, and ASf had lower (9–66%) AtUPM1 protein abundance (Supplementary Figures 2E,F).

### The *AtUPM1* overexpressors modulated nitrogen assimilatory enzymes

#### Nitrite reductase: gene expression, protein abundance, and enzyme activity

**Figure 2:**
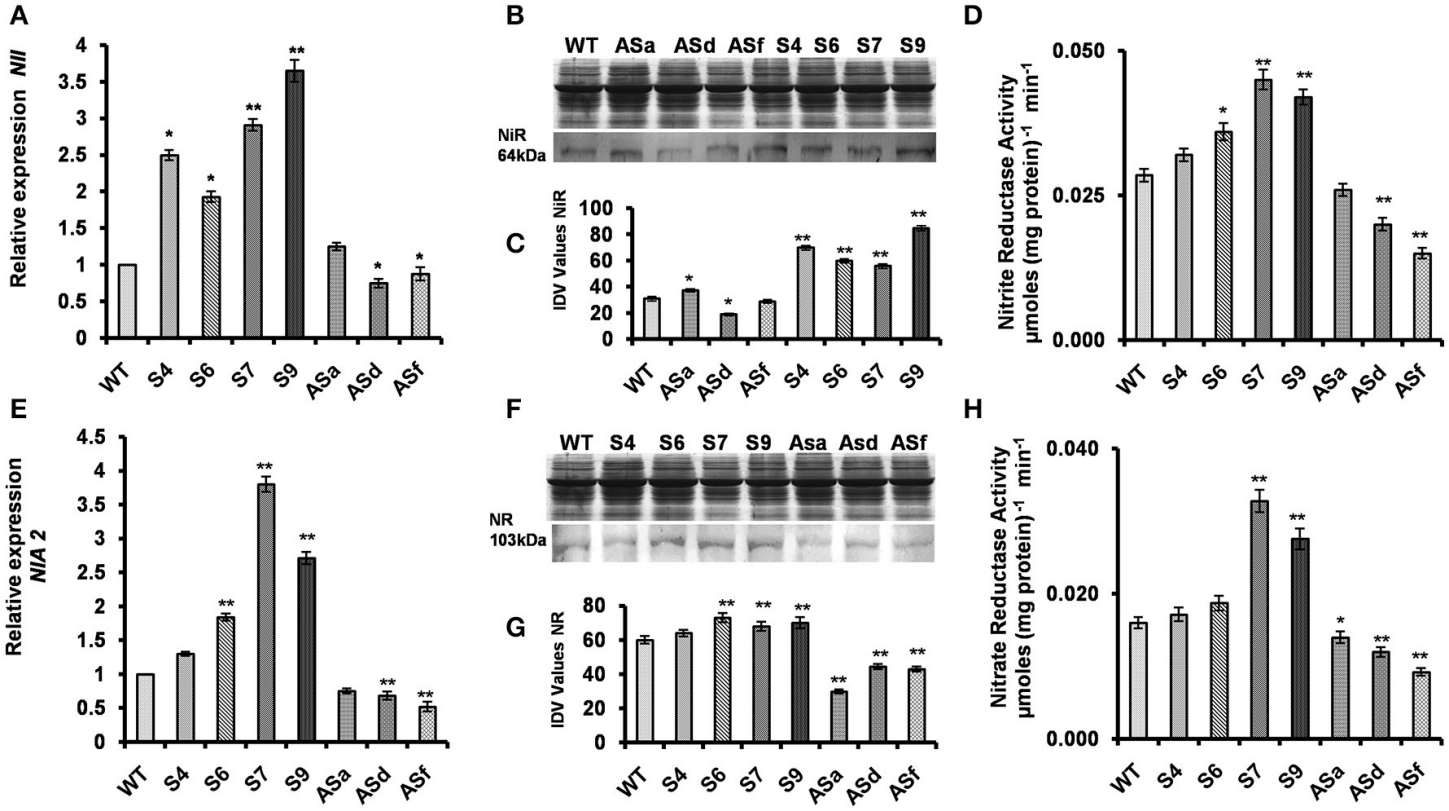
Modulation of nitrite reductase and nitrate reductase in the transgenics. The WT and *AtUPM1* overexpressor and antisense plants were grown photoperiodically (14 h L and 10 h D) in MS plates for 25 days under cool-white fluorescent light at 21°C. **(A)** The changes in gene expression of nitrite reductase (*NII*) in transgenics monitored by qRT PCR. **(B)** SDS PAGE (12.5%) of protein (20 μg) isolated from the WT and transgenic plants to check equal loading and the immunoblot of the plant protein to check the abundance of nitrite reductase (NiR). **(C)** The quantification of the NiR blot by densitometric analysis using Alpha Ease FC software. **(D)** The NiR enzyme activity of the WT and *AtUPM1* transgenic plants. **(E)** The changes in gene expression of nitrate reductase (*NIA2*) in transgenics monitored by qRT PCR. **(F)** SDS-PAGE (12.5%) of protein (20 μg) isolated from the WT and transgenic plants to check equal loading and the immunoblot of the plant protein to check the abundance of nitrate reductase (NR). **(G)** Quantification of the NR blot by densitometric analysis using Alpha Ease FC software. **(H)** NR enzyme activity of the WT and *AtUPM1* transgenic plants. Western blot data is an average of three independent replicates. qRT-PCR data are expressed as the mean ± SEM of three independent experiments performed in triplicate. For enzyme assay, the experiment was repeated three times and each datapoint is an average of 5 biological replicates (*n* = 5). Error bars, S.D. Asterisks indicate significant difference determined by Student's *t*-test compared to control (^*^P < 0.05, ^**^P < 0.01) IDV values represent integrated density values as calculated by the Alpha Ease FC software.

UPM1 is the first enzyme involved in the biosynthesis of siroheme, the prosthetic group of nitrite reductase (see section Introduction). Thus, we suggest that the overexpression of *AtUPM1* may lead to modulation of nitrite reductase. Figure 2A shows that the overexpression of *AtUPM1*results in increased *NiR* gene expression. As revealed by q-RT PCR, the S4, S6, S7, and S9 overexpressor lines had 2.5-, 1.93-, 2.91-, and 3.65-fold higher gene expression than the WT (Figure 2A). Further, the western blot analysis, of the same lines, shows an increased abundance of nitrite reductase by 125, 106, 87, and 174%, respectively (Figures 2B,C). On the other hand, the *antiUPM1* plants had reduced gene and protein expression of NiR. However, ASd and ASf lines had 29 to 12% lower gene expression than the WT (Figure 2A); this was accompanied by a decrease (38 and 10%) in the abundance of nitrite reductase (Figures 2B,C). The ASa line was a very weak transgenic line and did not have any significant impact on gene expression or protein abundance of NiR. To understand the efficacy of the increased gene and protein expression of *UPM1* involved in the biosynthesis of the cofactor siroheme, the nitrite reductase activity was monitored in WT and transgenic plants. The NiR activity in the transgenic lines S4, S6, S7, and S9 was higher by 14, 29, 60, and 50% than that of WT. On the other hand, the antisense plants had lower (8–46%) NiR activity than the WT (Figure 2D).

#### Nitrate reductase: gene expression, protein abundance, and enzyme activity

The substrate for chloroplastic NiR, ${\text{NO}}_{2}^{-}$, is generated by the cytoplasmic enzyme NR that utilizes ${\text{NO}}_{3}^{-}$ as its substrate. Therefore, the gene expression, the protein abundance and the enzymatic activity of NR were monitored in the WT and all the transgenic plants. As compared to WT, the transcript abundance of *NIA2* in *AtUPM1*overexpressors was higher by 30, 84, 280, and 170% in transgenic S4, S6, S7, and S9, respectively (Figure 2E). Similarly, the nitrate reductase protein abundance in S4, S6, S7, and S9 were higher than WT by 10, 20, 50, and 45% respectively (Figures 2F,G). In *antiUPM1* plants both gene expression and protein abundance of NR was substantially lower than WT. The enzyme activity of nitrate reductase increased in sense transgenic lines. The NR activity in S4, S6, S7, and S9 was 8, 19, 106, and 75% higher than in WT. On the contrary, in *anti UPM1* transgenic lines the NR activity was substantially lower (13–43%) than the WT (Figure 2H).

### Overexpression of UPM1 increased protein and pigment content

#### Protein content

**Figure 3:**
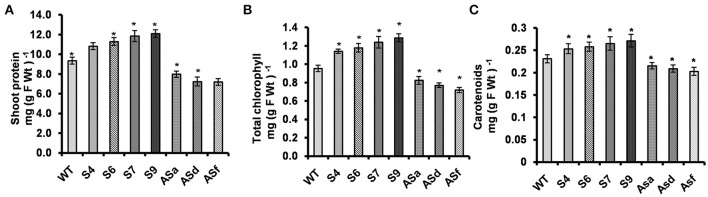
*AtUPM1* modulates the protein content, chlorophyll and the carotenoid content. The WT and *AtUPM1* overexpressor and antisense plants were grown photoperiodically (14 h L and 10 h D) in MS plates for 25 days under cool-white fluorescent light at 21°C. **(A)** Shoot protein content. **(B)** Shoot chlorophyll content. **(C)** Shoot carotenoid content. The experiments were repeated three times and each datapoint is an average of ten biological replicates for (f), (g) and (h). Error bars, S.D. Asterisks indicate significant difference determined by Student's t test compared to control (^*^P < 0.05, ^**^P < 0.01) IDV values represent integrated density values as calculated by the Alpha Ease FC software.

The total protein content of all the overexpressors (the sense plants) was higher than in the WT (Figure 3A): proteins in the shoots of *AtUPM1x* S4, S6, S7, and S9 were 15–30% higher. However, in the antisense plants, the protein content was lower (15–23%). The reason for this result is most likely due to changes in gene expression as well as protein abundance and the activity of nitrogen assimilation enzymes (NiR and NR): higher in the sense plants and lower in the antisense plants. We note that the lowering of these enzymes, in the antisense plants, may have been due to siroheme limitation.

#### Chlorophyll content

Increase in the total protein content in *AtUPM1x* plants (S4, S6, S7, and S9) was accompanied by higher chlorophyll content: 20–35% more than in the WT (Figure 3B). In contrast, the antisense plants (*antiUPM1*) had reduced (13–25%) and Chl content than in the WT.

#### Carotenoid content

We note that the *AtUPM1x* lines S4, S6, S7, and S9 had higher (10–15%) carotenoid content than the WT (Figure 3C). However, the antisense plants had reduced (7–12.5%) carotenoid content.

### *AtUPM1* modulates the expression of genes involved in chlorophyll biosynthesis

**Figure 4:**
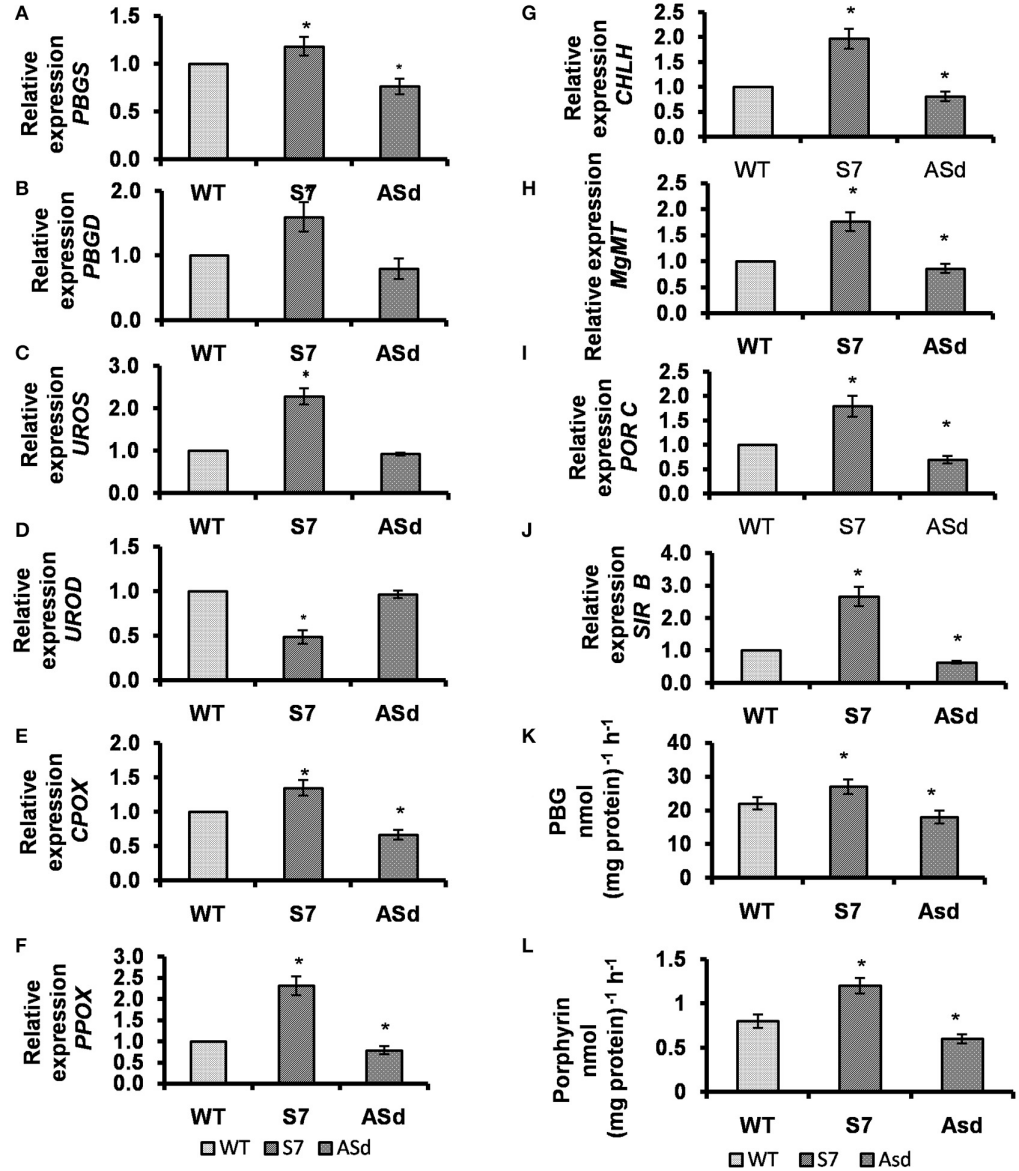
Analysis of the transcript abundance of nuclear genes encoding the enzymes involved in the tetrapyrrole biosynthesis. The relative gene expression of **(A)** *PBGS* **(B)** *PBGD* **(C)** *UROS* **(D)** *UROD* **(E)** *CPOX* **(F)** *PPOX* **(G)** *CHLH* **(H)** *MgMT* **(I)** *PORC* **(J)** *SIRB* **(K)** PBGS, and **(L)** PBGD enzyme activities in leaves of WT and transgenic plants. Transcript levels were calculated as relative expression (2^−ΔΔCT^) according to Livak and Schmittgen (2001) in comparison to WT and *Actin2* as a reference gene. Data are given as means and SD of three biological replicates. WT, *AtUPM1x* and *antiUPM1* transgenic plants were photoperiodically (14 h L and 10 h D) grown in MS plates for 25 days under cool-white fluorescent light at 21°C. Data are expressed as the mean ± SEM of three independent experiments performed in triplicate. Asterisks indicate significant difference determined by Student's t test compared to control (^*^P < 0.05).

Differences in Chl content of the transgenic plants, used in this study, led us to ask if *AtUPM1* expression can modulate the gene expression of the enzymes involved in Chl biosynthesis. For this purpose, we chose S7, an overexpressor having higher Chl content, and ASd, an underexpressor having lower Chl content. (We did not use ASf because it had multiple insertion of the transgene.) Using quantitative reverse transcription (qRT)-PCR, we analyzed the transcript abundance of enzymes involved in the biosynthesis of protoporphyrin IX and of Mg-tetrapyrrole. The gene expression of porphobilinogen synthase (*PBGS*), responsible for the synthesis of the pyrrole ring porphobilinogen (PBG), increased by 22% in S7, a sense plant; however, it decreased by 24% in ASd (Figure 4A). Further, the gene expression of PBG deaminase (PBGD) and uroporphyrinogen III synthase (UROS), involved in cyclization of four molecules of PBG to uroporphyrinogen III, was upregulated by 60 and 128% in the S7 line, and was downregulated by 24 and 20% in the underexpressor respectively (Figures 4B,C). In contrast, the gene expression of uroporphyrinogen III decarboxylase (*UROD*) (AT2G40490.1), responsible for decarboxylation of uroporphyrinogen III to coporporphyrinogen III, declined by ~50% in S7. However, in ASd, the transcript abundance of *UROD* was almost similar to that of the WT plant (Figure 4D).

In S7, a sense plant, the message abundance of coproporphyrinogen III oxidase (*CPOX*), which converts coproporphyrinogen III to protoporphyrinogen IX, was higher (35%) than in the WT, and lower (33%) in ASd, an antisense plant (Figure 4E). In the same vein, the gene expression of protoporphyrinogen IX oxidase (*PPOX*), responsible for the removal of six electrons from the macrocycle of protoporphyrinogen IX to form protoporphyrin IX, was higher (by 130%) in S7 than in the WT, and lower (by 22%) in Asd (Figure 4F).

To ascertain the impact of *UPM1* overexpression on the Mg–branch of tetrapyrrole biosynthesis, the gene expression of two selected enzymes involved in the conversion of porphyrin IX to chlorophyll was monitored: (1) Mg-chelatase, which is responsible for chelation of Mg to protoporphyrin IX moiety, which directs porphyrin to the Mg-branch of tetrapyrrole biosynthesis; we used only the CHLH moiety of this enzyme. (2) Mg-protoporphyrin IX: S-adenosyl methionine methyl transferase (*MgMT*), which converts Mg-Proto IX to Mg-protoporphyrin IX monomethyl ester (MPE). Using qRT, we showed that the message abundance of *CHLH* increased by 97% in S7, a sense plant and decreased by 20% in ASd, an antisense plant (Figure 4G). The gene expression of *MgMT* was higher by 76% in S7, and lower by 14% in ASd than the WT plant (Figure 4H). Further, for the Chl biosynthesis pathway, we observed that the gene expression of protochlorophyllide oxidoreductase C (*PORC*), a light-inducible gene responsible for photo-transformation of protochlorophyllide to chlorophyllide, increased by 79% in the overexpressor S7, and decreased by 31% in the underexpressor ASd (Figure 4I).

### The activities of chlorophyll biosynthetic enzymes

To ascertain if increased or decreased expression of the genes involved in Chl biosynthesis in the sense and antisense plants corresponds to changes in enzymatic activities, catalytic reactions of the following two important successive enzymes of Chl biosynthetic pathway were monitored in the transgenics.

*Porphobilinogen Synthase:* PBGS mediates the condensation of two molecules of ALA to yield the pyrrole moiety porphobilinogen (PBG) (Tanaka and Tanaka, 2007). In comparison with the WT, the PBGS activity increased by 23% in the S7 overexpressor, the sense plant, and decreased by 18% in ASd, the antisense plant (Figure 4K).

*Porphobilinogen Deaminase:* PBGD, in concert with uroporphyrinogen III synthase (UROS), converts four molecules of PBG to uroporphyrinogen III (Tanaka and Tanaka, 2007). As compared to that in the WT, the PBGD activity increased by 50% in S7 plants and decreased by 25% in ASd plants (Figure 4L).

### The gene expression of *AtSIRB*, the other known enzyme involved in siroheme biosynthesis, is co-regulated by *AtUPM1*

The abundance of message of sirohydrochlorin ferrochelatase (*AtSIRB*), an enzyme downstream of *UPM1* in the siroheme biosynthetic pathway, is found to be coregulated with *AtUPM1* in our study. Our experiments on the qRT-PCR showed that the overexpression of *AtUPM1* results in increased transcript abundance of *AtSIRB* in S7 by 2.5-fold. However, in ASd the message abundance decreased (Figure 4J).

### Chlorophyll a fluorescence, photosynthetic performance, and plant productivity of the transgenic plants

#### Chlorophyll *a* fluorescence

When a photosynthetic organism, kept in dark, is exposed to light, Chl fluorescence rises from a low minimum level (“O” level or Fo) to a higher maximum level (“P” level or Fm). The maximum primary photochemical efficiency of PSII was estimated from Fv/Fm, where Fv = Fm-Fo (Papageorgiou and Govindjee, 2004).

##### Fo

The minimal fluorescence, Fo, increased in S4, S6, S7, and S9 (sense lines) by18, 20, 21, and 25%, respectively over that of WT (Table 1). This increase in minimal fluorescence was mostly due to increase in the amount of Chl (Figure 3) in different transgenic lines. In the antisense lines (ASa and ASd) the Fo declined by 3–9% mostly due to their reduced Chl content.

**Table 1:** Chl *a* fluorescence parameters of WT and transgenic plants.

**Plant line**	**Chl** ***a*** **fluorescence**	
	**Fo**	**Fv/Fm**
WT	0.0472 ± 0.001	0.784 ± 0.011
S4	0.0556 ± 0.002	0.792 ± 0.011
S6	0.0566 ± 0.003	0.795 ± 0.010
S7	0.0572 ± 0.002	0.811 ± 0.012
S9	0.0589 ± 0.001	0.821 ± 0.014
Asa	0.0512 ± 0.002	0.761 ± 0.011
Asd	0.0483 ± 0.003	0.748 ± 0.013

*Leaves from wild-type and AtUPM1x and antiAtUPM1 plants were dark adapted for 20 min before measurement of their minimal fluorescence (Fo) and maximum primary photochemical efficiency (Fv/Fm) by a PAM 2100 fluorometer. The experiment was repeated three times, and the values are means ± SD (n = 5)*.

##### Fv/Fm ratio

The Fv/Fm ratio of the dark adapted plants reflects the potential quantum efficiency of PSII, which affects overall photosynthetic efficiency of plants. The overexpression lines had higher (0.80–0.82) and underexpressor plants had lower (0.75–0.76) Fv/Fm ratio than that of WT (0.78) (Table 1); this could imply better performance of the sense plants over the others.

##### Electron transport rate

**Figure 5:**
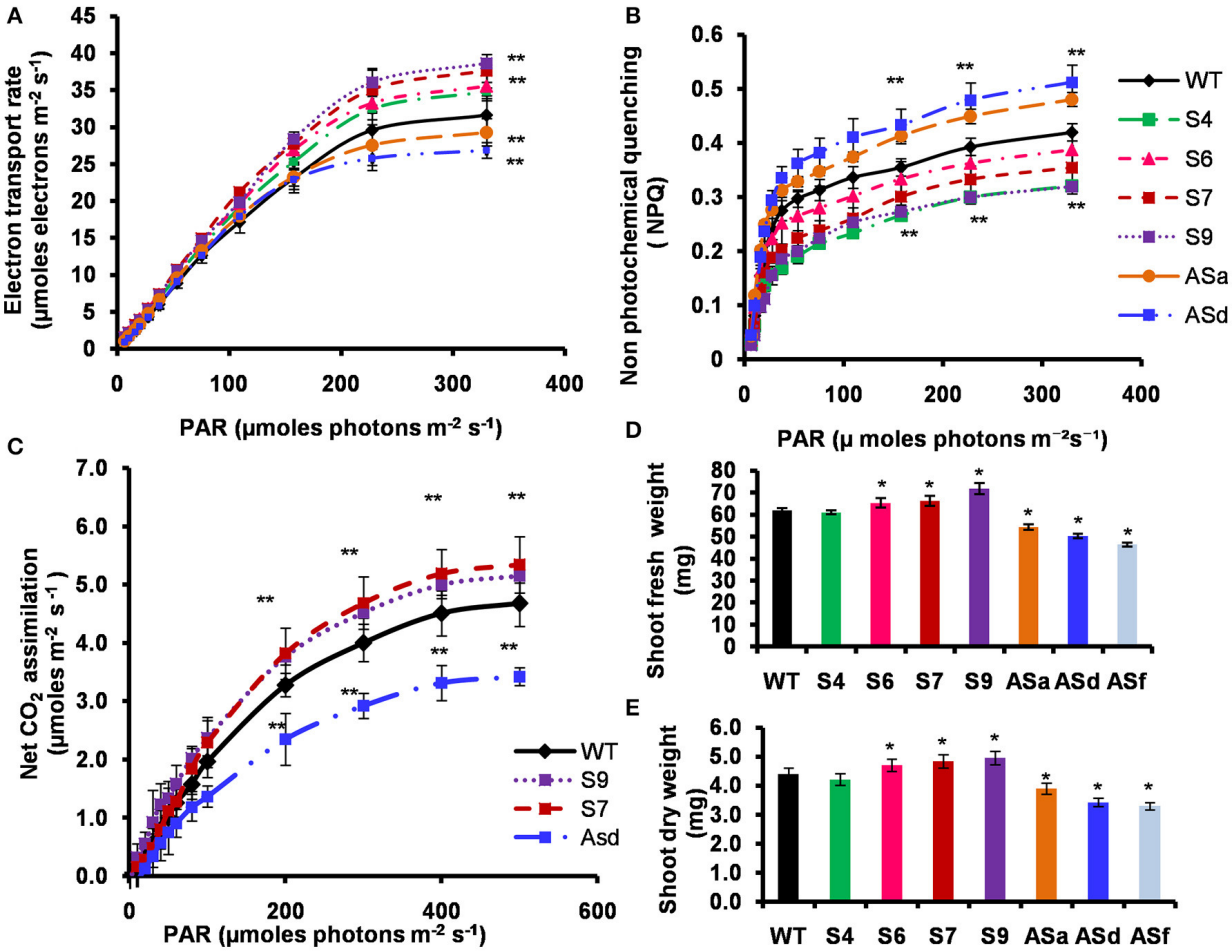
Light response curves of PSII-dependent electron transport, non-photochemical quenching (NPQ), net photosynthetic CO_2_ assimilation, shoot fresh weight and dry weight of the WT and transgenic plants. The WT and transgenic plants were grown photoperiodically (14 h L and 10 h D) in MS plates for 25 days under cool-white fluorescent light at 21°C for Chl *a* fluorescence measurements. For IRGA measurements, the plants were grown in similar photoperiodic conditions for 5 weeks in agropeat and vermiculite mixture under cool-white fluorescent light at 21°C. **(A)** PSII-dependent electron transport rates of leaves in limiting and saturating actinic light intensities measured using a PAM 2100 fluorometer (Walz). **(B)** Non-photochemical quenching (NPQ) of excited states of chlorophylls of leaves in limiting and saturating actinic light intensities measured using a PAM 2100 fluorometer (Walz). **(C)** Light response curve of CO_2_ assimilation rates measured by infra-red gas analyser. Net CO_2_ assimilation rates of attached leaves of wild-type and transgenic plants were monitored with an infrared gas analyzer (Walz GFS-3000 portable photosynthetic system) in ambient CO_2_ at different light intensities. Light-response curves were measured up to 500 μmol photons m^−2^ s^−1^ at 25°C. **(D)** Shoot fresh weight and **(E)** shoot dry weight of 25 days old MS grown plants under similar growth conditions. The experiments were repeated three times. Five biological replicates were taken for experiments **(A–C)**. Ten biological replicates were taken for **(D,E)**. Error bars, S.D. Asterisks indicate significant differences determined by Student's *t*-test compared to control (^*^P < 0.05, ^**^P < 0.01).

As expected, the electron transport rate (ETR), as inferred from fluorescence data (for details, see section Materials and Methods) increased in response to photosynthetic active radiation (PAR) (μmol photons m^2^ s^−1^) (Figure 5A). These light response curves demonstrate that electron transport rates (in μmol electrons m^−2^ s^−1^) for the transgenic lines under limiting light (10–50 μmol photons m^−2^ s^−1^), as well as under saturating light, were substantially higher than the WT. At the saturating light intensity, the S4, S6, S7, and S9 lines had 10–22% higher ETR than the WT. In antisense plants, ETR was downregulated by 16% (Figure 5A).

##### Non-photochemical quenching (NPQ)

The non-photochemical quenching (NPQ), of the excited state of chlorophyll *a*, is a good indicator of dissipation of excess light energy absorbed by plants, algae and cyanobacteria (Demmig-Adams et al., 2014). At saturating light intensity, the NPQ of several *AtUPM1x* transgenic lines was 8–24% lower than the WT. In contrast, in the antisense lines, the NPQ was higher (15–22%) than the WT (Figure 5B). These data clearly suggest that the sense plants would perform better than the antisense plants.

#### Carbon assimilation

Since the ETR was higher in the sense transgenic plants than the WT, we expected increased rates of photosynthesis. We monitored the light response curves of CO_2_ assimilation, and found that, in comparison to the WT plants, the sense transgenic lines had higher (12–14%) carbon assimilation rate, at the highest light intensity used (Figure 5C). However, in the antisense plants, the carbon assimilation rate at saturating light intensity declined by 27% (Figure 5C).

#### Fresh weight and dry weight of the shoots

Our data show that the sense transgenic lines grew better than the WT and had a higher biomass (Figures 5D,E). As compared to WT, the T3 generation of homozygous S7 and S9 transgenic lines had 10–16% higher fresh weight of the shoots (Figure 5D). Further, the S7 and S9 overexpressor plants had 10–12% higher dry weight than the WT plants (Figure 5E). On the other hand, the antisense plants had 12–25% lower fresh weight and dry weight than the WT.

### Responses of *AtUPM1x* and *antiUPM1* transgenic plants to nitrogen deficiency

The results obtained under normal growth conditions (see section Materials and Methods) demonstrated that *AtUPM1*overexpressor sense plants had better N assimilatory capacity and the underexpressor antisense plants had lower N-utilization ability (Figure 2). Therefore, the transgenic plants were tested under nitrogen starvation conditions to assess their tolerance to N-deficiency. As the representative lines for these studies, we chose the sense S7 as it had the highest nitrate reductase and nitrite reductase activities, and antisense ASd as it had the lowest activities of these enzymes.

### Plant phenotype under nitrogen starvation conditions

**Figure 6:**
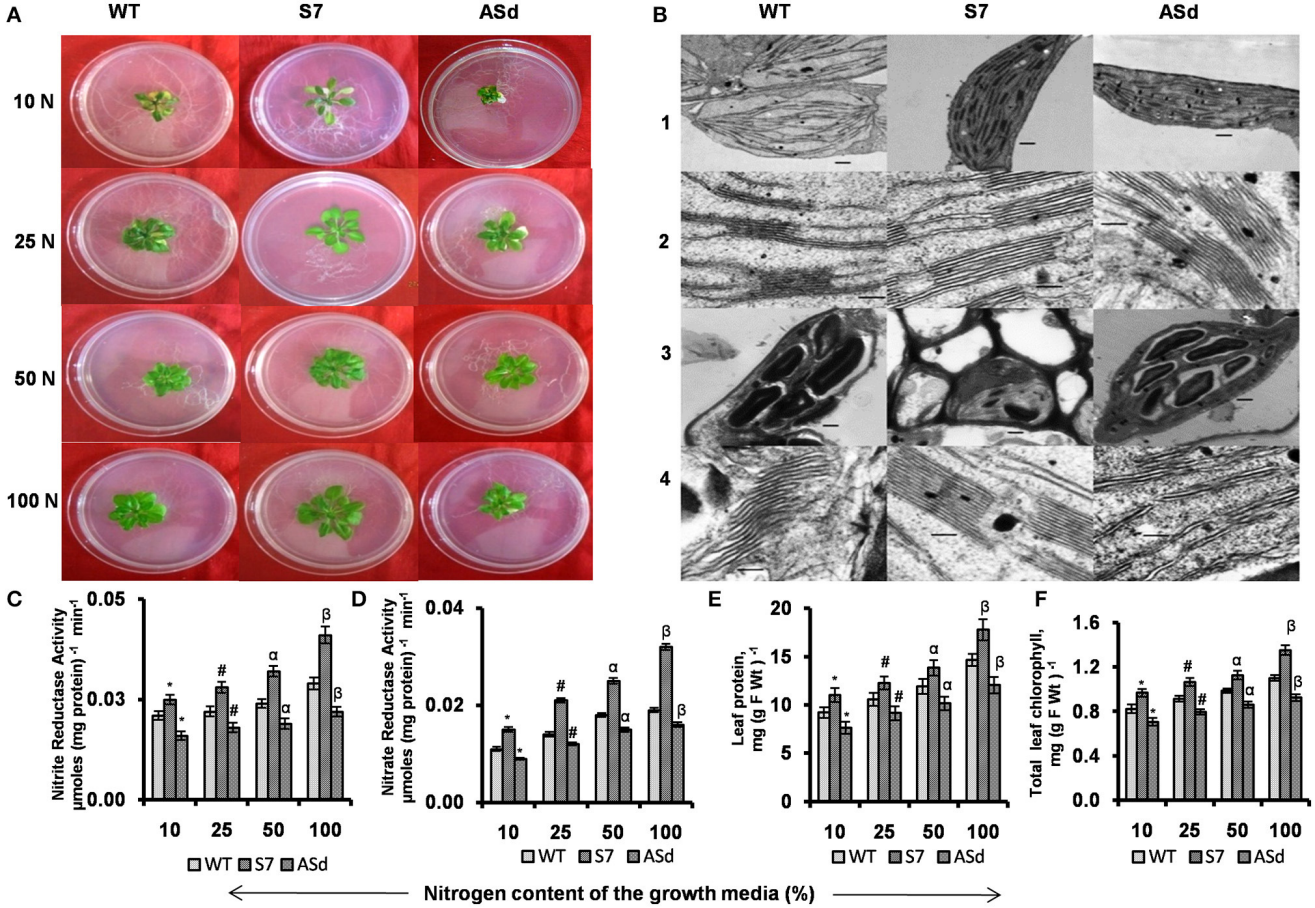
Plant phenotype, nitrogen assimilatory enzymatic activities, protein and chlorophyll content and ultrastructure of chloroplast of WT and transgenic plants grown in N-sufficient and N-deficient growth media. WT and transgenic plants were grown under normal MS medium for 10 days and subsequently transferred for 15 days to different N-deficient (10% N, 25% N, 50% N) or N-sufficient (100% N) conditions. Nitrogen concentration for 100 N was 5 mM. Plants were grown under 14 h L and10 h D photoperiod under cool-white-fluorescent light at 21°C. After 15 days plants were taken for analysis. **(A)** Representative phenotypes of WT and *AtUPM1* transgenic plants. **(B)** Representative transmission electron micrographs at (1 and 3) 3000X and (2 and 4) 25000X magnification. The scale bars (1 and 3) and (2 and 4) represent 500 nm and 100 nm respectively. The third leaf was sampled for electron microscopy. Panels 1 and 2 represent ultra-structures in 100% N; panel 3 and 4 represent the ultra-structures in 10% N. **(C)** Nitrite reductase (NiR) activity. **(D)** Nitrate reductase (NR) activity. **(E)** Leaf protein content. **(F)** Leaf chlorophyll content. The experiments were repeated three times. Five biological replicates were taken for **(C,D)**. Each data point is an average of 10 biological replicates for **(E,F)**. Error bars, S.D. Different symbols denote significant difference in the parameters determined by Student's *t*-test compared to respective control under different conditions (^*^P < 0.05, ^#^*P* < 0.05, ^α^*P* < 0.05, ^β^*P* < 0.05).

Under N deficiency (10% N), the phenotype of WT plants was different from that of the overexpressors. The *AtUPM1x* plants were greener and bigger in size than those of the WT and had fewer pale leaves. The antisense plants were smaller in size and there was a marked discoloration of leaves (Figure 6A and Supplementary Figure 3).

### ${\text{NO}}_{3}^{-}$ assimilatory enzyme activities were higher in *AtUPM1x* plants under N-deficient condition

Under N-deficient growth conditions (10–50% N), the overexpressor sense plants had 17–23% higher NiR activity and the antisense plants had 20–23% lower activity than the WT (Figure 6C). The substrate for NiR, i.e., ${\text{NO}}_{2}^{-}$ is produced by NR, the first enzyme of the nitrogen assimilation pathway. The NR activity of the sense plants was higher than that of the WT under N starvation conditions. When grown under 10–50% N, the sense transgenic (S7) plants had 36–38% higher NR activity, whereas antisense transgenic (ASd) had lower (16–18%) activity of the same enzyme than the WT plants (Figure 6D).

### The overexpressors retained more protein and chlorophyll during nitrogen starvation

For the following studies, the plants (WT, S7, and ASd) were first grown in N sufficient (100% N) (normal MS) conditions and then transferred to N-deficient (50, 25, and 10% N) growth conditions, as described under Material and Methods. We measured both the protein and the chlorophyll content in these samples under different nitrogen conditions.

Our results on plants grown under 10, 25, 50, and 100% N showed that the protein content was higher by 17–20% in the overexpressor plants, and lower by 15–18% in the antisense plants, as compared to that of WT (Figure 6E).

Our results on the chlorophyll content showed a decline in the WT and the transgenics due to N deficiency (Figure 6F).Under 50–90% N-deficient conditions, the S7 plants had higher (15–17%) and the antisense plants had lower (13–15%) chlorophyll content than the WT.

### Chlorophyll *a* fluorescence under nitrogen starvation

#### The Fo fluorescence

Under both the N sufficient and N-starvation conditions, the minimal fluorescence Fo of overexpressors was higher than in the WT. However, the antisense plants had lower Fo than the WT (Table 2).

**Table 2:** Chl *a* fluorescence parameters of WT and transgenic plants grown in N starvation conditions.

**Plant type**	**N**_**2**_ **content (%)**							
	**100**		**50**		**25**		**10**	
	**Fo**	**Fv/Fm**	**Fo**	**Fv/Fm**	**Fo**	**Fv/Fm**	**Fo**	**Fv/Fm**
WT	0.0613 ± 0.003	0.763 ± 0.006	0.0565 ± 0.004	0.694 ± 0.011	0.0528 ± 0.004	0.688 ± 0.009	0.0515 ± 0.004	0.68 ± 0.006
S7	0.0691 ± 0.003	0.783 ± 0.015	0.0672 ± 0.005	0.754 ± 0.023	0.0627 ± 0.004	0.738 ± 0.029	0.0606 ± 0.002	0.725 ± 0.011
ASd	0.0569 ± 0.003	0.729 ± 0.003	0.0513 ± 0.004	0.657 ± 0.003	0.0474 ± 0.003	0.662 ± 0.006	0.0458 ± 0.003	0.657 ± 0.015

*Leaves from wild-type and AtUPM1x and antiAtUPM1 plants grown under N starvation conditions as described in Material and Method were dark adapted for 20 min before measurement of their minimal fluorescence (Fo) and maximum primary photochemical efficiency (Fv/Fm) by a PAM 2100 fluorometer. The experiment was repeated three times, and the values are means ± SD (n = 5)*.

#### The Fv/Fm ratio

Our results show that the overexpression line S7 had a higher Fv/Fm ratio under control and under all N deficiency conditions than the WT. On the other hand, the antisense plants had a lower ratio than the WT (Table 2).

#### Electron transport rate, as inferred from Chl *a* fluorescence

**Figure 7:**
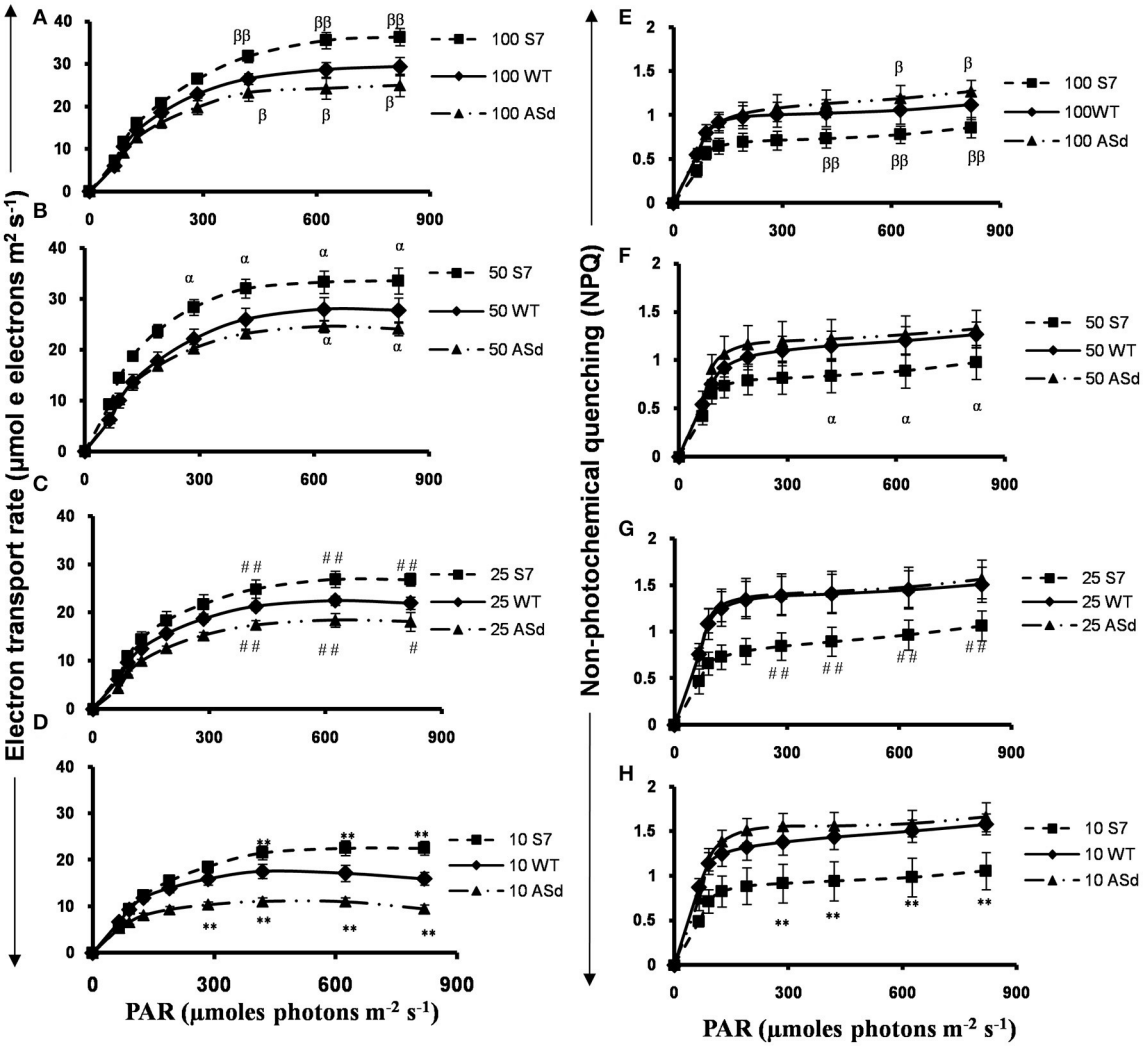
Pulse Amplitude Modulated (PAM) Chl *a* fluorescence measurements in WT and *AtUPM1* overexpressor and antisense plants grown in N-sufficient and N-deficient growth media. WT and *AtUPM1* transgenic plants were grown in normal MS medium for 10 days and subsequently transferred for 15 days to different N-deficient or N-sufficient conditions (10, 25, 50, and 100 N correspond to percentage of nitrogen in the growth media, respectively). Nitrogen concentration for 100 N was 5 mM. Plants were grown in 21°C under 14 h Land 10 h D photoperiod under cool-white-fluorescent light at 21°C. After 15 days of growth in N-deficient media plants were taken for measurements of electron transport rate and non-photochemical quenching. Electron transport rates in plants in **(A)** 100% N, **(B)** 50% N, **(C)** 25% N, **(D)** 10% N. Non-photochemical quenching of excited states of chlorophyll **(E)** 100% N, **(F)** 50% N, **(G)** 25% N, and **(H)** 10% N. The experiments were repeated three times with similar results. Each data point is an average of five biological replicates. Error bars, S.D. Different symbols denote significant difference in the parameters determined by Student's *t*-test compared to respective control. (^#^*P* < 0.05, ^α^*P* < 0.05, ^β^*P* < 0.05: ^**^P < 0.01, ^##^*P* < 0.01, and ^ββ^*P* < 0.01).

The electron transport rate (ETR) was estimated under the nutrient stress conditions for the WT, sense (*AtUPM1x*) and antisense (*antiUPM1*) plants. The sense plants had a higher estimated electron transport rate at saturating light intensities: at 10, 25, 50, and 100% nitrogen, the rates were 23–41% higher than in the WT. However, in the antisense plants, the estimated ETR was lower (15–40%) than in the WT under N-starvation conditions (Figures 7A–D).

#### Non-photochemical quenching (NPQ) under nitrogen starvation

As expected, the NPQ of the excited state of chlorophyll *a* increased with increasing light intensity. We note that N starvation (10% N) increased NPQ in the WT by 50%; however, under 10, 25, and 50% N, the sense (*AtUPM1x*) plants had lower (upto 33%) NPQ, whereas, the antisense (*antiUPM1*) plants had higher NPQ (upto 13%) than the WT (Figure 7E through Figure 7H).

### Transmission electron microscopy (TEM) of the WT and *AtUPM1* transgenics under nitrogen starvation

At 100% N, which is the optimum nutrient condition, the WT, the *AtUPM1x* (S7) and the *antiUPM1* (ASd) are similar in their granal organization and thylakoid architecture, as visualized by TEM (Figure 6B). However, major differences were observed when the plants were grown under N starvation. We observed that the WT and the antisense plants accumulate more starch granules than the *AtUPM1x* plants. The grana lamella of the WT was more destacked than that of the S7 plants. However, in the ASd plants, thylakoids were partially swollen and almost completely destacked under N-deficient media (Figure 6B).

### The *AtUPM1* modulates sulfur assimilation

#### Gene expression and protein abundance of SiR

**Figure 8:**
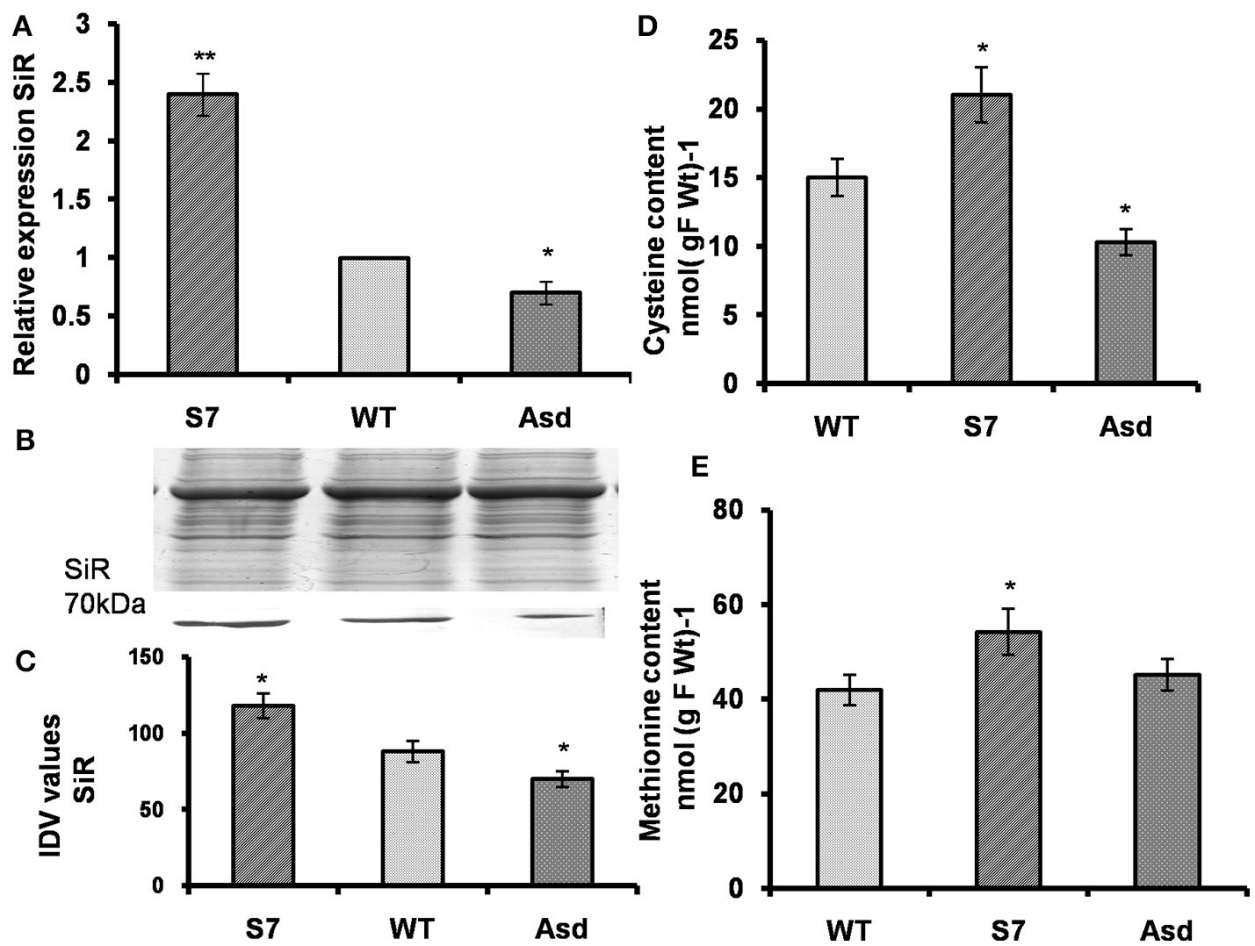
*AtUPM1* modulates sulfur assimilation. The WT and transgenic plants were grown photoperiodically (14h L and 10 h D) in MS plates for 25 days under cool-white fluorescent light at 21°C. **(A)** The changes in gene expression of sulfite reductase (*SiR*) in transgenics monitored by qRT PCR. **(B)** SDS-PAGE (12.5%) of protein (20 μg) isolated from the WT and transgenic plants to check equal loading and immunoblot of plant protein to check the abundance of sulfite reductase (SiR). **(C)** Quantification of the SiR blot by densitometric analysis using Alpha Ease FC software. **(D)** Cysteine content of leaves. **(E)** Methionine content of leaves. Western blot was repeated three times and data is an average of three independent replicates. qRT-PCR data are expressed as the mean ± SEM of three independent experiments performed in triplicate. Each data point for **(D,E)** is an average of three biological replicates, performed three times. Error bars, S.D. Asterisks indicate significant difference determined by Student's *t*-test compared to control (^*^P < 0.05, ^**^P < 0.01). IDV values represent integrated density values as calculated by the Alpha Ease FC software.

We note that siroheme is the prosthetic group of SiR, just as it is of NiR; it mediates ferredoxin dependent reduction of ${\text{SO}}_{3}^{2-}$ (Murphy et al., 1974). Here, we have monitored the impact of *AtUPM1* overexpression on *SiR* gene expression as well as on protein abundance in WT, and in both sense (S7) and antisense (Asd) plants. The q-RT PCR results showed that, as compared to the WT, the sense line S7 had 2.5-fold higher, and the antisense line Asd had 30% lower expression of *SiR* (Figure 8A). Further, as revealed from the western blot analysis, the relative amount of SiR, as compared to the wild type, increased by 34% in the sense plant and decreased by 21% in the antisense plant (Figures 8B,C).

#### The overexpressors had more cysteine and methionine content than the WT and antisense plants

Among S containing amino-acids, the cysteine content was 40% higher and 31% lower, in *AtUPM1x*-S7 and *antiUPM1-*Asd transgenic lines than the WT (Figure 8D). In addition, the total methionine content increased by 29% and decreased by 10% in the sense and antisense plants, respectively (Figure 8E).

#### Response of *AtUPM1x* and *antiUPM1* transgenic plants to sulfur deficiency

Our results showed that *AtUPM1* sense plants had higher, and the antisense plants had lower *SiR* gene expression and SiR protein (Figure 8A). This must have led to better S assimilation resulting in higher cysteine and methionine content in the sense plants. We discuss below our studies on S deficiency in WT, sense and antisense plants.

#### Plant phenotype

**Figure 9:**
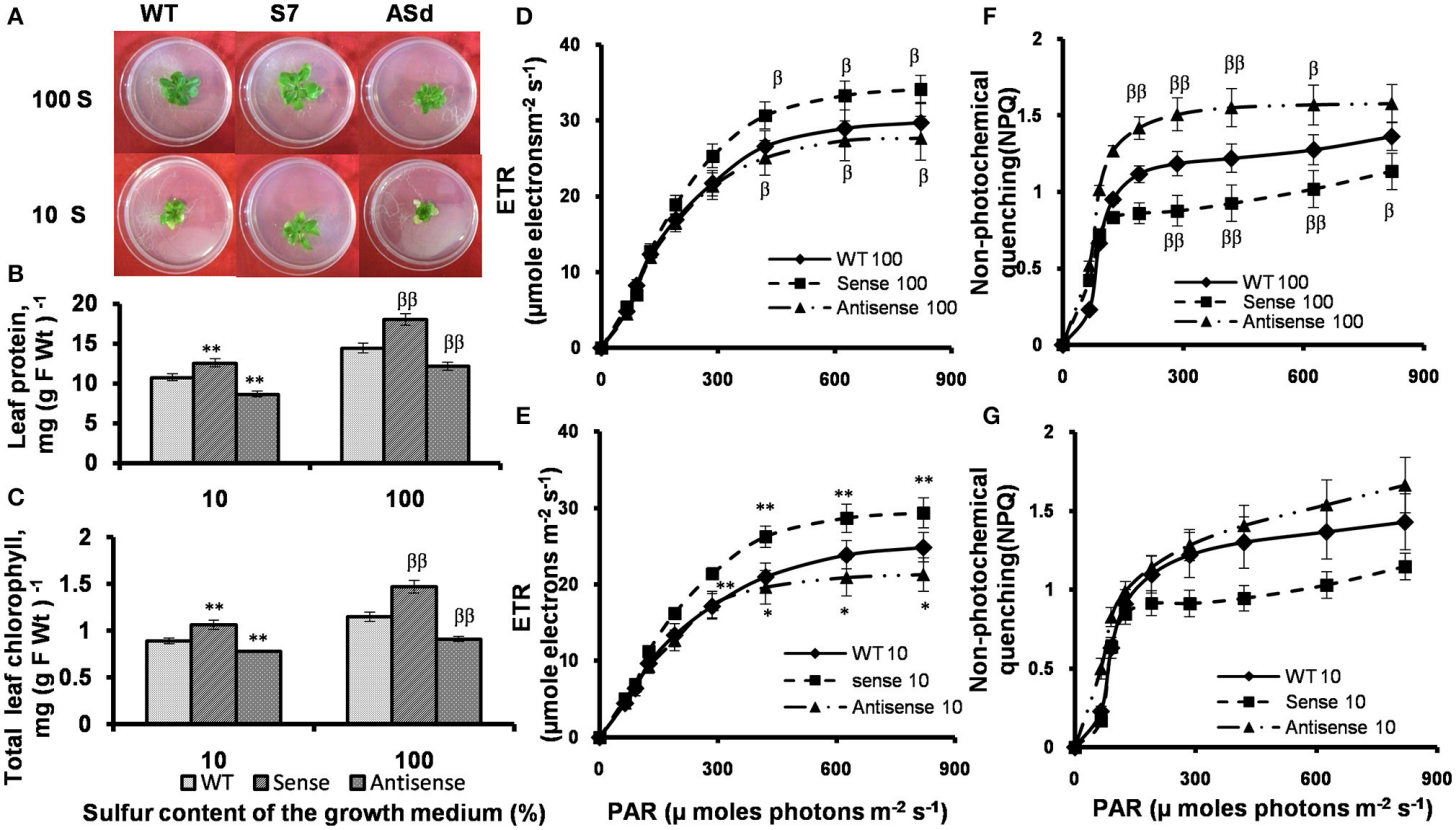
Plant phenotype, protein and chlorophyll content and PAM Chl *a* fluorescence measurements in WT and transgenic plants grown in S-sufficient and S-deficient growth media. WT and transgenic plants were grown under normal MS medium for 10 days and subsequently transferred for 15 days to S-deficient (10% S) or S-sufficient (100% S) conditions. S concentration for 100 S was 2 mM. Plants were grown under 14 h Land 10 h D photoperiod under cool-white-fluorescent light at 21°C. After 15 days plants were taken for analysis. **(A)** Representative phenotypes of WT and *AtUPM1* transgenic plants. **(B)** Leaf protein content. **(C)** Leaf chlorophyll content. Electron transport rates in plants in **(D)** 100% S and **(E)** 10% S. Non-photochemical quenching of excited states of chlorophyll **(F)** 100% S and **(G)** 10% S. The experiments were repeated three times with similar results. Each data point is an average of 10 biological replicates for **(B,C)** and five biological replicates for **(D–G)**. Error bars, S.D. Different symbols denote significant difference in the parameters determined by Student's t test compared to the respective control under different conditions. (^*^P < 0.05, ^β^*P* < 0.05: ^**^P < 0.01, ^ββ^*P* < 0.01).

The WT, sense line S7 and antisense line ASd were first grown in S sufficient (100% S) conditions, and then transferred to S-deficient (10% S) conditions. In S deficient conditions, the *AtUPM1x* plants were comparatively greener and looked bigger than the WT and antisense plants (Figure 9A).

#### The overexpressors (sense plants) retained more protein and Chl under S-starvation

The total soluble leaf protein was estimated in S-sufficient and S-deficient growth conditions. The *AtUPM1x* had 25% higher and antisense plants had 17% lower protein content than the WT under optimal (100% S) growth condition (Figure 9B). Under S starvation (10% S) the sense plants had a higher (17%) and antisense plants had lower (20%) protein content than the WT (Figure 9B). We note that under S-deficient conditions, the sense plants had higher (19%), and the antisense plants lower (13%) chlorophyll content than the WT plants (Figure 9C).

#### Chlorophyll *a* fluorescence under S-starvation

To understand the impact of S starvation on photosynthesis, the photosynthetic performance, as inferred from chlorophyll *a* fluorescence measurements of WT and transgenic plants was studied under S-sufficient and S-deficient growth conditions.

##### Fo

In S-deficient media, the Fo, the minimum fluorescence, of the sense plants was 10% higher and that of antisense plants was 20% lower than the WT (Table 3).

**Table 3:** Chl *a* fluorescence parameters of WT and transgenic plants grown in S starvation conditions.

**Plant type**	**S content (%)**			
	**100**		**10**	
	**Fo**	**Fv/Fm**	**Fo**	**Fv/Fm**
WT	0.063 ± 0.002	0.804 ± 0.012	0.058 ± 0.003	0.751 ± 0.021
S7	0.064 ± 0.002	0.806 ± 0.009	0.063 ± 0.003	0.787 ± 0.013
ASd	0.061 ± 0.004	0.761 ± 0.007	0.047 ± 0.001	0.699 ± 0.017

*Leaves from wild-type and AtUPM1x and antiAtUPM1 plants grown under S starvation conditions as described in Material and Methods were dark adapted for 20 min before measurement of their minimal fluorescence (Fo) and maximum primary photochemical efficiency (Fv/Fm) by a PAM 2100 fluorometer. The experiment was repeated three times, and the values are means ± SD (n = 5)*.

**Table 4:** List of primers used for qRT PCR.

**Gene**	**Forward primer**	**Reverse primer**
*UPM1*	CCCAAACCCATATAAACGACAAG	GTAAGGTTTCTCACTGGTCATTA
*NII*	AGCCAGTTCTGCGGACAAGC	CGTCAGCACCCTCGACTGGC
*NIA2*	GCTAGTCTGCGCGGGGAACC	GGACACCACGCCACACGGAG
*SIR B*	TCGCAGTTCTTAGTGAACCTCTCTTAC	GTGATCCTCTTCTCCTCCTTCCT
*PBGS*	GCAAAAGAGCTACGTAGGCC	CTCTTGGAAGGCAGCTCTCT
*PBGD*	CCAGAAGAGCTTTGTTTCCG	TCACAAAGTCGTTCTCACGC
*UROS*	CAAGTCCTGGTTTCTCTGGG	ATGAGGAGCAATGGAGCAAT
*UROD*	TGAGCTTGGACTGGACTGTG	GCCCCAAGTTGAGAATGTGT
*CPOX*	GAGCTCCGAGGCAATGGT	GGATCAGGTTTAACGACCGA
*PPOX*	AAGCCTAATTCGACCGATCC	CCTAAGGCTACACCAGCGAC
*CHLH*	TTCAGTAGAGTTGATTTCAGTGGC	TGGAAGCAAGTTAAATGCAGG
*MgMT*	GCGTATCTACACTCGGAGGC	CATTGGAACAGCTTCGATGA
*POR C*	TCAGGAGTGTATTGGAGTTGGA	AAGCTTCTCGCTAACCTCCC
*ACTIN2*	CTTGAAGTATCCTATTGAGCATGGTGTT	CGGGAGAGTTAAAGGTCTCAAACATGA

##### Fv/Fm

In *AtUPM1x* plants, the Fv/Fm ratio, an indirect measure of quantum yield of PSII, was marginally higher (5%) than that of WT grown under sulfur starvation. On the other hand, the Fv/Fm ratio declined by 7% in *antiUPM1* plants (Table 3).

##### Electron transport rate (ETR)

Under optimal growth conditions, and under saturating light, the ETR, as inferred from chlorophyll *a* fluorescence data, of the sense plant was 15% higher and that of antisense plants was 7% lower than that in the WT. Under S starvation condition, the ETR of *AtUPM1x* plants under saturating light intensities was 19% higher than in the WT, whereas in *antiUPM1* plants, the ETR decreased by 15% (Figures 9D,E).

##### Non-photochemical quenching (NPQ)

The NPQ of chlorophyll excited state increased in response to higher light intensities. In contrast to ETR, under normal growth condition, in saturating light intensity, the NPQ of the sense plant was 20% lower than that in the WT plant, and that of antisense plants was about 16% higher. Under sulfur starvation, the NPQ of *AtUPM1x* plants was 21% lower than that of WT, whereas in *antiUPM1* plants, the same increased by 18% (Figures 9F,G).

## Discussion

In the present study, genetic manipulation of tetrapyrrole biosynthesis pathway, which governs carbon, nitrogen and sulfur assimilation, has given us an insight on the co-regulation of these three different but inter-dependent biological processes. The first tetrapyrrole of Chl biosynthesis pathway, i.e., uroporphyrinogen III is shown in this paper to be partially hijacked *via* over-expression of uroporphyrinogen III methyl transferase (*AtUPM1*) in *A. thaliana* to synthesize more siroheme, the cofactor required for activity of NiR and SiR. The Southern blot analysis of *AtUPM1* in WT and transgenic plants revealed that *A. thaliana* has a single copy of the gene. The sense lines S7, S8, and S9 and antisense lines ASa and Asd had the single integration of the transgene (Supplementary Figure 2). Overexpression of *AtUPM1* under the control of constitutive _35_S promoter resulted in increased expression of the gene and the gene product in the sense plants and their reduced expression in antisense plants. We found that the abundance of the message of sirohydrochlorin ferrochelatase (*SIRB*), which functions downstream to UPM1 in the siroheme biosynthesis pathway, was coregulated with *AtUPM1. SIRB* expression increased in *At UPM1x* plants and decreased in antisense plants.Increased abundance of UPM1 and SIRB, enzymes working in siroheme biosynthesis pathway, is likely to enhance siroheme content in *AtUPM1x* transgenic plants. In the same vein, in the antisense plants the siroheme content is likely to be lower.

### Modulation of N assimilation by UPM1

Siroheme biosynthesis takes place in chloroplasts as all the enzymes, involved in siroheme biosynthesis, occur in the stroma (Tripathy et al., 2010). Increased amount of cofactor siroheme, present in chloroplasts, might have induced the enhancement of the nuclear genes expression of *NII* coding for NiR by retrograde signaling (Oelmüller et al., 1986; Mochizuki et al., 2001; Schlicke et al., 2014; Zhou et al., 2015; Pattanayak and Tripathy, 2016). However, the exact chemical nature of the molecule(s) responsible for retrograde signaling is still unknown. A report suggests that the overexpression of *GmCnx1*, responsible for increasing the biosynthesis of molybdenum cofactor, results in higher NR activity in transgenic soybean plants (Zhou et al., 2015). This demonstrates that specific cofactors may modulate the gene expression of the apoproteins. We suggest that increased expression of nitrite reductase (*NII)*, led to the increase in NiR protein abundance and enzymatic activity in different overexpressor lines, used in our study. In contrast, reduced siroheme accumulation in chloroplasts of antisense plants could have given a negative feedback for lower nuclear *NII* gene expression than that in WT plants. Lower *NII* gene expression, NIR protein abundance and enzymatic activity were clearly observed in different antisense lines (Figure 2). The substrate of NiR is produced by NR that reduces ${\text{NO}}_{3}^{-}$ to ${\text{NO}}_{2}^{-}$ in the cytoplasm. The *NIA2* has predominant expression in *A. thaliana* (Wilkinson and Crawford, 1991). The other gene *NIA1* encodes the cytosolic minor isoform of NR and contributes about 10% of the total activity in shoots (Wilkinson and Crawford, 1991). The comodulation of *NIA2* expression in *AtUPM1*overexpressors resulted in upregulation of protein abundance and activity of NR (Figure 2). This must have replenished the ${\text{NO}}_{2}^{-}$ consumed by elevated NiR activity in the sense plants (Seligman et al., 2008; Zhao et al., 2009). Our results (Figure 2) demonstrate that modulation of genes involved in the synthesis of the cofactor of one of the enzymes involved in N assimilation regulates the gene expression of other enzymes of N assimilatory pathway. The prosthetic group siroheme could be a limiting factor in N assimilation and its improved synthesis in *AtUPM1x* plants may be crucial in NiR assembly and activity.

A significant finding in our study (this paper) was the increase of total protein content in the *AtUPM1* overexpressors (Figure 3). This increase could be attributed to higher N assimilatory enzymatic activities in the plant cell. The higher total protein content of *AtUPM11x* transgenic plants implies an increase in both structural and functional protein components of the cell. The overexpression of uroporphyrinogen III methyl transferase is expected to lead to an increase in siroheme levels, which must help in N assimilation in the form of enhanced ${\text{NH}}_{4}^{+}$ generation (Figure 1). We suggest that the ammonium generated must have been efficiently utilized by the overexpressors for amino acid and protein synthesis (Figure 3). Therefore no ${\text{NH}}_{4}^{+}$ toxicity was observed in overexpressors. Similarly, the efficient reduction of sulfite to sulfide by sulfite reductase must have resulted in an increase in the levels of S containing amino acids i.e., cysteine and methionine. Therefore no sulfide-induced toxicity was observed in the overexpressors (sense plants) (Figure 8).

Earlier studies with over-expression of *NiR* apoprotein under the control of 35S promotor in *N. plumbaginifolia* and *A. thaliana* did not show any increase in total protein content in the transgenics (Crété et al., 1997; Takahashi et al., 2001). Similarly, other enzymes involved in N assimilation i.e., NR, GS2, GOGAT or nitrate transporters in different plants did not result in increased protein content of plants (Vincentz and Caboche, 1991; Kozaki and Takeba, 1996; Ferrario-Méry et al., 2002). The NR has long been considered to be the rate-limiting step in nitrate assimilation and is subjected to transcriptional and post translational regulation by environmental factors such as light, CO_2_, oxygen availability as well as the redox state of the cell (Huber et al., 1994; Kaiser and Huber, 1994). Efforts to improve nitrogen utilization by over-expressing *NIA2* coding for NR in tobacco and potato did not result in significant change in protein or biomass accumulation (Quilleré et al., 1994; Djennane et al., 2002). Improvement in nitrogen utilization via manipulation of plastidic glutamine synthetase (*GS2*) and Fd-GOGAT (*GLU1*) has, however, met with some limited success. Transgenic tobacco plants with over-expression of *GS2* were shown to have rather higher photorespiration that did not have increased protein content per g fresh weight (Kozaki and Takeba, 1996; Ferrario-Méry et al., 2002; Bao A. et al., 2014). There are conflicting reports of overexpression of cytosolic GS1. The overexpression of root *PsGS1* in tobacco was shown to have increased protein content, demonstrating the role of secondary metabolic processes in enhancing nitrogen utilization (Oliveira et al., 2002). However, overexpression of shoot cytosolic glutamine synthatase (*OsGS1.1)* and root cytosolic glutamine synthetase (*OsGS1.2*) in rice resulted in poor growth phenotype and yield (Bao Y. et al., 2014). Further, plants over-expressing the *NpNRT2.1* gene encoding high affinity nitrate transporter were similar in protein content to their WT level, despite an increase in the ${\text{NO}}_{3}^{-}$ uptake indicating that higher ${\text{NO}}_{3}^{-}$ influx may not necessarily lead to concomitant improvement in nitrate utilization (Fraisier et al., 2000).

Our results demonstrate that stimulation of cofactor synthesis rather than apoprotein accumulation results in improved nitrogen utilization, and increased protein content and higher biomass (Figures 2, 5). This approach for the development of nitrogen use efficient transgenic plants seems to be much more promising.

### Modulation of chlorophyll biosynthesis by UPM1

Overexpression of *UPM1* is expected to result in increased diversion of uroporphyrinogen III to siroheme biosynthesis. Therefore, we expected a reduced Chl content in *AtUPM1x* plants. On the contrary, Chl content increased in UPM1 overexpressors, and decreased in the underexpressors (Figure 3). This was due to increase in total protein content of *AtUPM1x* transgenic plants that must have contributed to a general enhancement in the abundance of enzymes involved in Chl biosynthesis.

Among Chl biosynthetic genes, other than *UROD*, the transcript abundance of *PBGS, PBGD, UROS, CPOX, PPOX* I, involved in the synthesis of protoporphyrin IX, was upregulated in the sense plants and down regulated in the antisense plants. In the same vein, the activities of tetrapyrrole biosynthetic enzymes increased in the overexpressors and decreased in the antisense plants (Figure 4). The *UROD* expression was lower in sense plants and was relatively higher in antisense plants (Figure 4) possibly because of decreased or increased availability of the substrate uroporphyrinogen III. This reveals a retrograde signaling by the plastidic tetrapyrrole to the nucleus for the regulation of *UROD*. Further experiments are needed to ascertain the steady state concentration of uroporphyrinogen III in WT, sense and antisense plants inside and outside the chloroplast to elucidate the role of tetrapyrroles in retrograde signaling. Our results suggest that Protoporphyrin IX in sense plants was diverted to the Mg-tetrapyrrole biosynthesis pathway due to increased expression of *CHLH, MgMT*, and *PORC* (Figure 4). These suggest that even if one of the genes in the Chl biosynthesis pathway is downregulated, it can be suitably compensated by the upregulation of other genes leading to increased Chl biosynthesis. It is quite possible that UROD is not a limiting enzyme for Chl biosynthesis.

We have previously observed co-regulation of gene and protein expression of enzymes involved in tetrapyrrole biosynthesis where overexpression of light-inducible protochlorophyllide oxidoreductase C (*PORC*) or chlorophyllide *a* oxygenase (*CAO*) was shown to co-modulate other genes of tetrapyrrole biosynthesis and photosynthesis (Pattanayak and Tripathy, 2002, 2011; Biswal et al., 2012). Earlier observations also demonstrated that overexpression of other Chl biosynthesis pathway genes, i.e., *CHLM* and *CHLG*, co-modulate the gene expression of several other tetrapyrrole biosynthetic genes (Alawady and Grimm, 2005; Shalygo et al., 2009). Similarly, increased Chl *b* synthesis delays senescence resulting in the retention of the message abundance of several Chl biosynthetic enzymes (Sakuraba et al., 2012). These demonstrate the existence of regulatory network among the genes coding for the enzymes involved in a metabolic chain reaction.

### Impact of increased chlorophyll and protein content on photosynthesis and plant productivity

Chl *a* fluorescence is often used as a non-invasive signature of photosynthesis (Strasser and Srivastava, 1995; Papageorgiou and Govindjee, 2004). Increased Chl content coupled with enhanced protein content led to a small increase in Fo in the sense plants. Further, decreased Chl and protein content of antisense plants resulted in reduced Fo (Table 1). As compared to that in WT, the maximum primary photochemical efficiency of PSII (as inferred from Fv/Fm) in dark-adapted leaves was higher in the sense plants and lower in antisense plants (Table 1). Increase in Fv/Fm suggests higher quantum efficiency of photosynthesis in the overexpressors.

As expected, Electron Transport Rates (ETR), calculated from yield parameters of PAM fluorometry (Genty et al., 1989), saturated at lower light intensity (350 μmol photons m^−2^ s^−1^) from plants grown in low light (80 μmol photons m^−2^ s^−1^). The ETR values at limiting light intensities (up to 80 μmol photons m^−2^ s^−1^) in *AtUPM1x*plants were higher than that of WT suggesting a larger light-harvesting antenna size in the sense plants, mostly due to increased Chl content. For the calculations of ETR, as described in the section Materials and Methods (Schreiber et al., 1995), a fixed absorption of 0.84 is used, whereas the overexpressors have higher (Chl) and in all likelihood would have slightly higher absorption than 0.84, and thus calculated ETR for sense plants is likely to be higher. *AtUPM1x* plants had higher Chl and protein and therefore recorded elevated ETR than that of WT plants in saturating light intensities. Antisense plants had reduced Chl and protein and therefore their ETR was lower than that of WT.

The NPQ is a measure of heat dissipation and a combined total for the combination of photo-protective mechanisms, state 1 and state 2 transition quenching, and photo-inhibition and photo-damage (Demmig et al., 1987; Demmig-Adams et al., 2014). When compared to the WT, NPQ was lower in the sense plants and higher in the antisense plants. This demonstrates that absorbed light energy was better utilized in photochemical reactions in the sense plants and had reduced utilization in the antisense plants. This resulted in higher ETR in the sense plants and lower ETR in the antisense plants.

Our data, presented in this paper clearly show that in response to *UPM1* overexpression the light-saturated photosynthetic carbon assimilation on a leaf area basis was significantly higher than in the WT (Figure 5). This corresponds well to our ETR measurements. We have expressed the carbon assimilation rates on leaf area basis and not on Chl basis since photosynthetic CO_2_ assimilation is not always proportional to Chl content (Yoshida, 1972). Our data (Figure 3) demonstrate that stimulation of siroheme biosynthetic pathway increases Chl and protein content which results in higher photosynthesis and biomass. Conversely, suppression of siroheme biosynthesis pathway led to reduced protein and Chl content that resulted in lower ETR, CO_2_ assimilation and decreased biomass.

The above findings are in agreement with a previous report from our laboratory where over-expression of CAO, that increased total Chl and Chl *b* content, also enhanced electron transport, C assimilation and biomass accumulation (Biswal et al., 2012). In another report, PSII photosynthetic efficiency, carbon assimilation and dry matter accumulation increased in plants over-expressing sedoheptulose-bis-phosphatase enzyme (Lefebvre et al., 2005). Similarly, transgenic tobacco plants expressing a bifunctional cyanobacterial fructose-1,6 /sedoheptulose-1,7-bisphosphatase in chloroplasts had higher photosynthesis and dry matter production (Miyagawa et al., 2001). In our laboratory, Kandoi et al. have previously shown that the overexpression of maize phosphoenolpyruvate carboxylase (PEPC) in *A. thaliana* resulted in increased photosynthesis and biomass (Kandoi et al., 2016). *PEPC* in C4 carbon metabolism also plays a crucial role in the nitrogen assimilation (Shi et al., 2015). Our results, presented in this paper, demonstrate that photosynthetic capacity per unit leaf area and plant dry matter could be increased in *Arabidopsis thaliana* by overexpressing a single chloroplastic enzyme UPM1, involved in siroheme biosynthesis. Our results further demonstrate that modulation of N and S assimilation by genetic manipulation of siroheme biosynthesis pathway could increase C assimilation and plant productivity.

### *AtUPM1* overexpression protects plants from nitrogen deficiency

When challenged with N deficiency, the sense plants performed better and antisense plants were worse than WT (Figure 6). As compared to WT, the NR and NiR activities were always higher in sense plants and lower in antisense plants in N-deficient environment; further, there was higher protein and Chl content in *AtUPM1x* plants, over that in WT plants, in N-depleted conditions. It is well known that light energy absorbed by Chl is used to drive photosynthesis (photochemistry) and excess energy can be dissipated as heat or it can be re-emitted as light i.e., as Chl *a* fluorescence. These three processes compete with each other such that an increase in efficiency of one results in a decrease in the yield of the other two (Baker, 2008). Hence, by measuring the yield of chlorophyll *a* fluorescence, information about changes in the efficiency of photochemistry and heat dissipation can be gained (Demmig-Adams and Adams, 2006; Kalaji et al., 2012). In our research, photosynthetic ETR was always higher than the WT in sense plants under 50-90% N starvation conditions. However, antisense plants had much lower ETR than the WT in N-deficient conditions. Under N starvation conditions, NPQ was lower than in the WT in sense and higher in antisense plants, demonstrating better energy utilization in the overexpressors (the sense plants) (Figures 7F,G).

The co-modulation of carbon and nitrogen assimilation is important for enhancement of nitrogen use capacity of plants (McAllister et al., 2012). The carbohydrate catabolic degradation leads to the generation of C skeletons that serve as precursors to amino acids such as alanine, serine, glutamate, and aspartate. Thus C and N metabolisms are tightly interlinked for plant adaptive response to any nutrient stress (Rufty et al., 1988; Doncheva et al., 2001; Bi et al., 2007). The starch content is known to increase under N starvation conditions, which is not due to increased starch synthesis, but rather due to lower N mobilization capacity in N deficient plants (Huber et al., 1989). The increase in starch granules in the electron micrographs of chloroplasts of WT and antisense plants and their absence from that of sense plants in Ndeficient (10% N) conditions (Figure 6B) demonstrates that *AtUPM1* overexpression could potentially make plants tolerant to N-starvation.

N-starvation leads to reduction in total fatty acids (Gaude et al., 2007) content leading to alteration in chloroplast ultrastructure. Galactolipids, associated with the chloroplast membrane system, are essential for chloroplast development. N limitation causes a decrease in MGDG that leads to defective chloroplast development and fewer thylakoid membranes (Jarvis et al., 2000; Gaude et al., 2007). Our results demonstrate that the organization of thylakoids was affected and that they did not stack properly in N-deficient WT and in antisense plants. This could be partly due to reduced MGDG content, reduced chlorophyll and pigment protein complexes responsible for grana stacking.

### *AtUPM1* regulates sulfur assimilation

Like NiR, SiR contains siroheme as its cofactor for 6 electron reduction of sulfite to sulfide (Murphy et al., 1974). Therefore, the increased presence of siroheme in the *UPM1* overexpressors resulted in the upregulation of *SiR* message abundance, possibly via retrograde signaling from the chloroplast to the nucleus. Consequently, the SiR protein abundance increased in *UPM1x* plants. We suggest that in the chloroplast of *AtUPM1x* plants, enhanced assembly of SiR holoenzyme efficiently must have reduced sulfite to sulfide. Cysteine synthesis from serine is known to be limited by sulfide availability. We suggest that in *AtUPM1x* plants, the reduction of sulfite to sulfide may have increased due to increased siroheme synthesis and *SiR* expression. This could possibly stimulate the synthesis of S-containing aminoacids cysteine and methionine leading to increased protein synthesis.

### *AtUPM1* overexpressors are tolerant to sulfur starvation

Sulfate assimilation is tightly coordinated with the carbon and nitrogen metabolism (Koprivova et al., 2000; Nakayama et al., 2000; Kopriva et al., 2002; Leustek, 2002). Sulfate is taken up and reduced to sulfite before being assimilated (Leustek and Saito, 1999). Although plants augment sulfate assimilation from the soil in S- deficient environment, plant growth and development is negatively affected when sulfur is low.

Our study demonstrates that when grown for 15 days in S-deficient medium (10% S), WT and antisense plants were deficient in protein and Chl and looked pale-green. Under S starvation, the protein content in antisense plants was lower than in WT *A. thaliana*, likely due to reduced synthesis of S containing amino acids. In the sense plants, the higher protein content may have been due to increased synthesis of cysteine and methionine. Higher availability of amino acids, including cysteine and methionine, must have increased the protein content of sense plants, which must have contributed to higher Chl content than in the WT and antisense plants in S deficient environment.

In optimal growth medium (100% S), which had increased Chl and protein content, photosynthetic rates were higher in the *AtUPM1x* plants than that in WT. In contrast, due to decreased Chl and protein content in *antiUPM1* plants, their photosynthetic efficiency was low. Further, in antisense plants, the estimated ETR was higher in *AtUPM1x* and lower in antisense plants in normal as well as S-deficient media in different light intensities. Finally, the NPQ of overexpressors (the sense plants) was lower than that in WT plants contributing to the advantage in their performance.

## Concluding remarks

In conclusion, the most pronounced outcome (or the highlight) of the present study is the increased protein content of *AtUPM1* overexpressors (the sense plants). Further, our study suggests that it is not the apoprotein (nitrite reductase or sulfite reductase), but the cofactor (siroheme) that limits the nitrogen and sulfur utilization. Therefore, the modulation of siroheme biosynthesis pathway is a potential target for improved N, S, and C assimilation and increased crop productivity. This approach could be potentially used to increase protein content of crop plants that could grow in low soil N and S environment with minimal application of fertilizers.

## Author contributions

BT designed the experiments: SG performed the experiments and analyzed the data: Paper was written by BT and SG.

### Conflict of interest statement

The authors declare that the research was conducted in the absence of any commercial or financial relationships that could be construed as a potential conflict of interest.

## Supplementary material

Supplementary Figure 1 Schematic representation of vector construction: Ligation of *AtUPM1* in sense and antisense orientation with linearized modified pCAMBIA 1304 vector having 35 S promoter and –Ω- translational enhancer cassette and *npt* for kanamycin selection.

Supplementary Figure 2 Confirmation and characterization of transgenics. The WT and *AtUPM1* overexpressor and antisense plants were grown photoperiodically (14 h L and 10 h D) in MS plates for 25 days under cool-white fluorescent light at 21°C. **(A)** PCR amplification of the genomic DNA with 35S internal forward and *AtUPM1* specific reverse primers for overexpressor (sense) plants. **(B)** PCR amplification of the genomic DNA with 35S internal forward and *AtUPM1* forward primers for antisense plants. **(C)** Southern blot of PCR positive transformed T3 lines to check for the copy number of gene integration. **(D)** qRT PCR of the T4 homozygous plants to check the relative expression of *AtUPM1* in WT and transgenic plants. **(E)** SDS PAGE (12.5%) of protein (20 μg) isolated from WT and transgenic plants to check equal loading and the immunoblot of the plant protein to check the abundance of AtUPM1. **(F)** The quantification of the AtUPM1 immunoblot using Alpha Ease FC software. IDV values represent integrated density values as calculated by the Alpha Ease FC software. Western blot data is an average of three independent replicates. qRT-PCR data are expressed as the mean ± SEM of three independent experiments performed in triplicate.Error bars, S.D. Asterisks indicate significant difference determined by Student's t test compared to control (^*^*P* < 0.05, ^**^*P* < 0.01).

Supplementary Figure 3 Phenotype of WT and transgenic plants grown vertically in square plates in N sufficient and deficient media. *Arabidopsis thaliana* seeds were directly germinated and grown for 3 weeks in square plates as described in Material and Methods. After 3 weeks of growth in N deficient (10% N) medium the antisense plants perished.
