# Corrigendum: Alleviation of Nitrogen and Sulfur Deficiency and Enhancement of Photosynthesis in *Arabidopsis thaliana* by Overexpression of Uroporphyrinogen III Methyltransferase (*UPM1*)

**DOI:** 10.3389/fpls.2018.01365

**Published:** 2018-10-04

**Authors:** Sampurna Garai, Baishnab C. Tripathy

**Affiliations:** School of Life Sciences, Jawaharlal Nehru University, New Delhi, India

**Keywords:** carbon assimilation, electron transport, nitrogen utilization efficiency, nitrogen deficiency, photosynthesis, siroheme, sulfur deficiency, uroporphyrinogen III methyltransferase1 (UPM1)

In the original article, we neglected to include the funder DST-PURSE, Government of India to Baishnab C Tripathy. The authors apologize for this error and state that this does not change the scientific conclusions of the article in any way.

The original article has been updated.

## Conflict of interest statement

The authors declare that the research was conducted in the absence of any commercial or financial relationships that could be construed as a potential conflict of interest.

