# Supplementary figures and images for: Alleviation of Nitrogen and Sulfur Deficiency and Enhancement of Photosynthesis in Arabidopsis thaliana by Overexpression of Uroporphyrinogen III Methyltransferase (UPM1)
Source: Front Plant Sci. 2018 Jan 23;8:2265. doi: 10.3389/fpls.2017.02265 (PMC5810253; doi:10.3389/fpls.2017.02265)

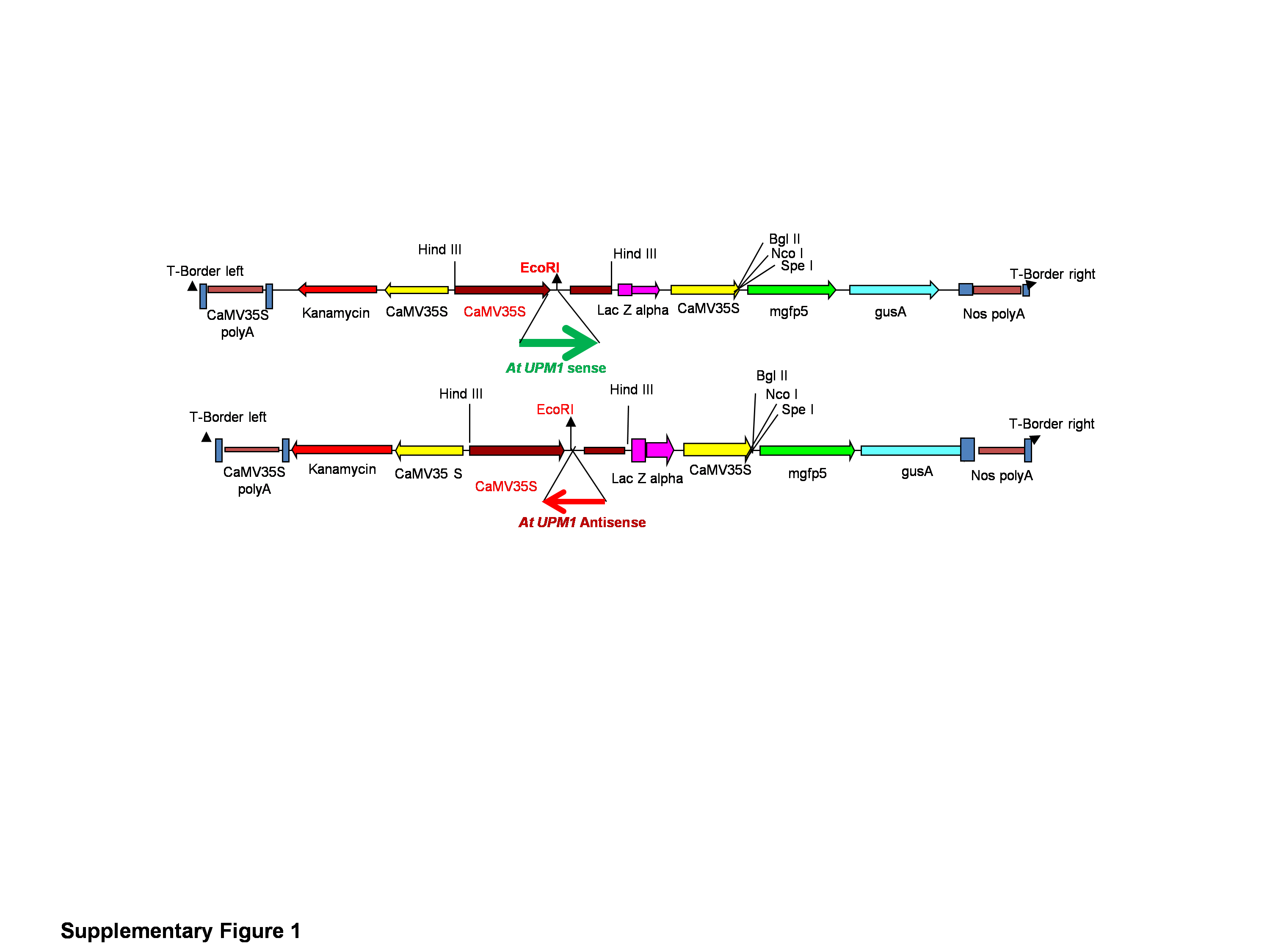

Supplement: Supplementary Figure 1 — Schematic representation of vector construction: Ligation of AtUPM1 in sense and antisense orientation with linearized modified pCAMBIA 1304 vector having 35 S promoter and –Ω- translational enhancer cassette and npt for kanamycin selection. [file Image1.TIF]

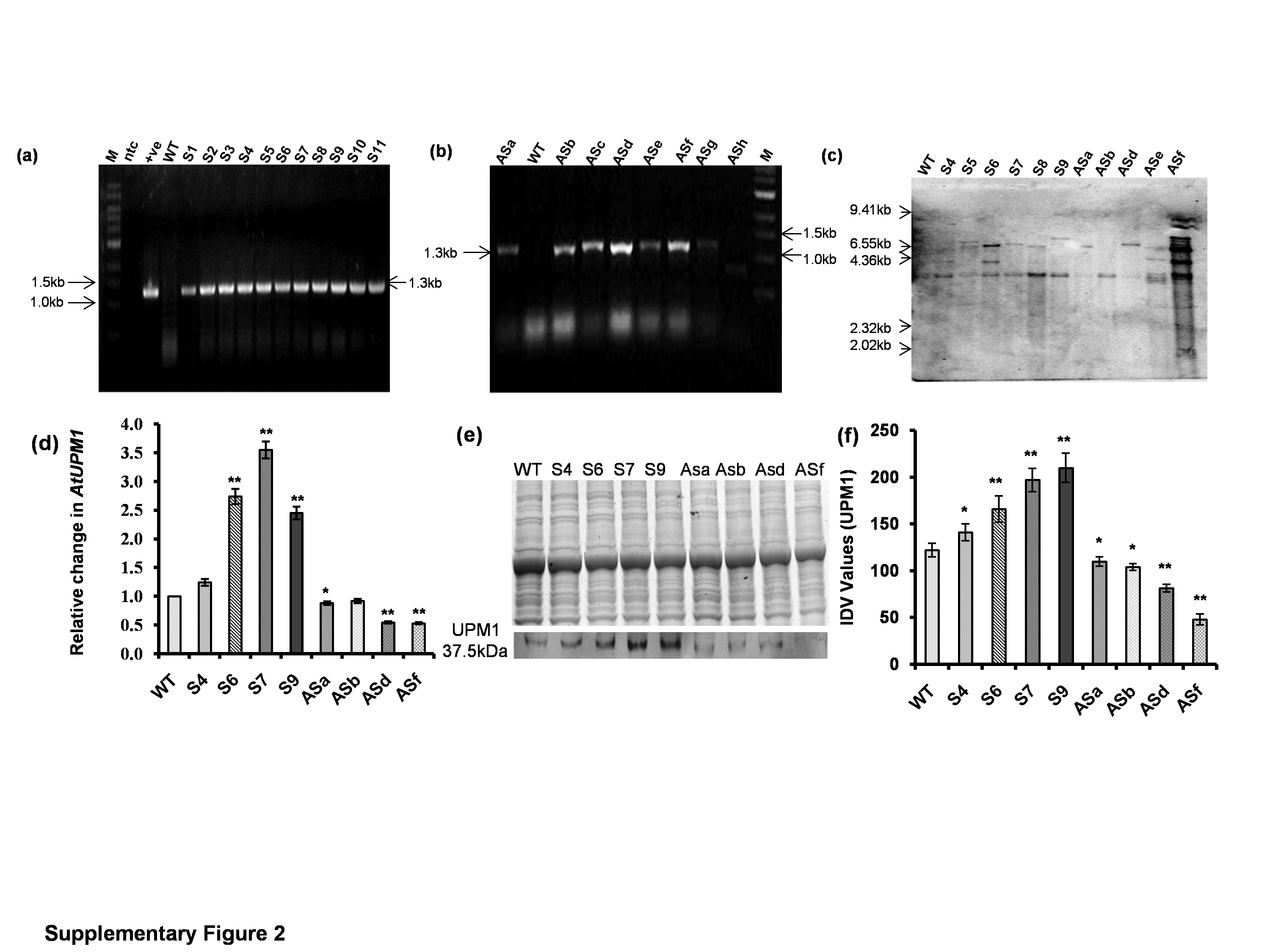

Supplement: Supplementary Figure 2 — Confirmation and characterization of transgenics. The WT and AtUPM1 overexpressor and antisense plants were grown photoperiodically (14 h L and 10 h D) in MS plates for 25 days under cool-white fluorescent light at 21°C. (A) PCR amplification of the genomic DNA with 35S internal forward and AtUPM1 specific reverse primers for overexpressor (sense) plants. (B) PCR amplification of the genomic DNA with 35S internal forward and AtUPM1 forward primers for antisense plants. (C) Southern blot of PCR positive transformed T3 lines to check for the copy number of gene integration. (D) qRT PCR of the T4 homozygous plants to check the relative expression of AtUPM1 in WT and transgenic plants. (E) SDS PAGE (12.5%) of protein (20 μg) isolated from WT and transgenic plants to check equal loading and the immunoblot of the plant protein to check the abundance of AtUPM1. (F) The quantification of the AtUPM1 immunoblot using Alpha Ease FC software. IDV values represent integrated density values as calculated by the Alpha Ease FC software. Western blot data is an average of three independent replicates. qRT-PCR data are expressed as the mean ± SEM of three independent experiments performed in triplicate.Error bars, S.D. Asterisks indicate significant difference determined by Student's t test compared to control (*P < 0.05, **P < 0.01). [file Image2.TIF]

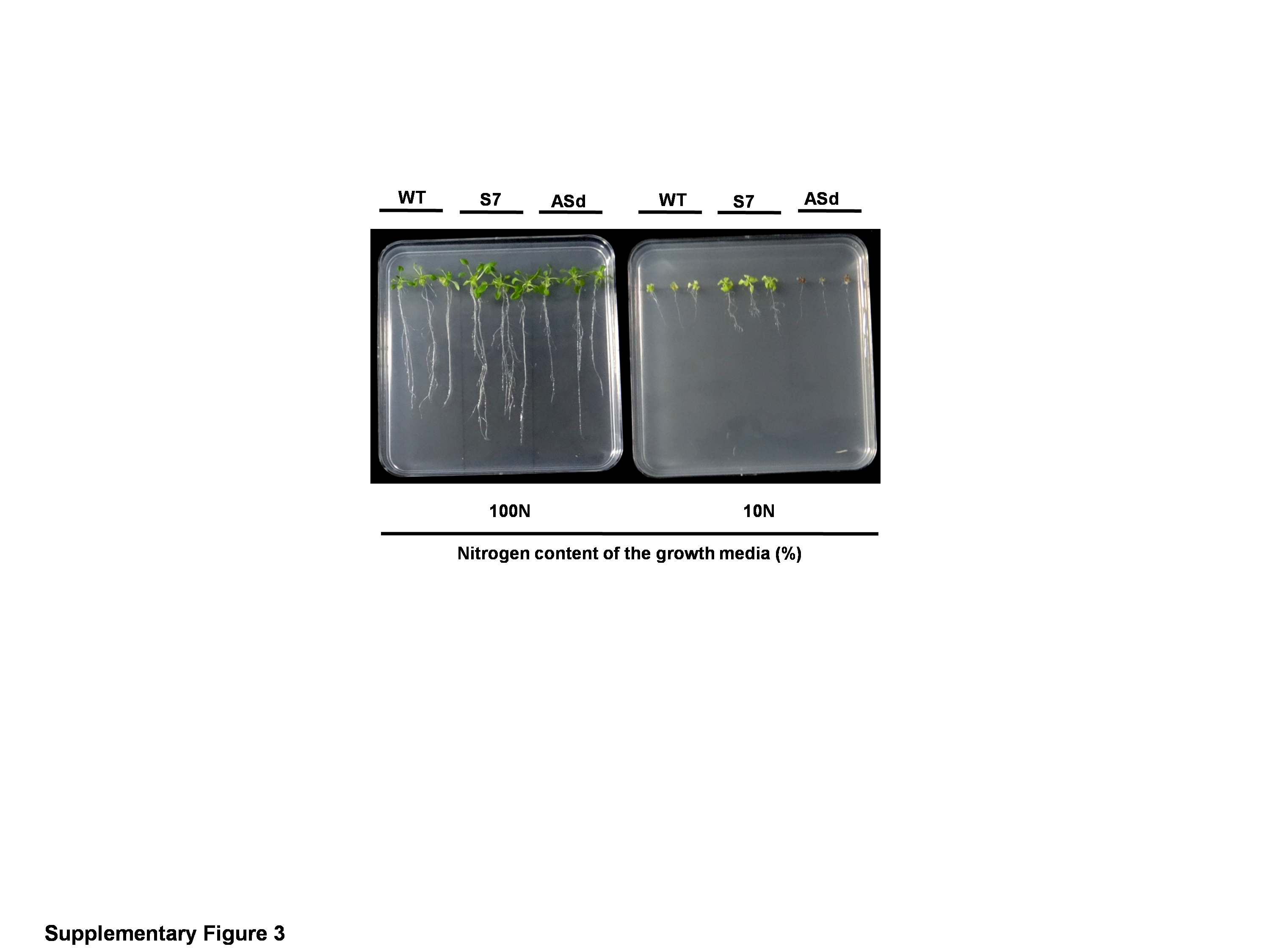

Supplement: Supplementary Figure 3 — Phenotype of WT and transgenic plants grown vertically in square plates in N sufficient and deficient media. Arabidopsis thaliana seeds were directly germinated and grown for 3 weeks in square plates as described in Material and Methods. After 3 weeks of growth in N deficient (10% N) medium the antisense plants perished. [file Image3.TIF]
